# *miR-22* promotes stem cell traits via activating Wnt/β-catenin signaling in cutaneous squamous cell carcinoma

**DOI:** 10.1038/s41388-021-01973-5

**Published:** 2021-08-03

**Authors:** Shukai Yuan, Peitao Zhang, Liqi Wen, Shikai Jia, Yufan Wu, Zhenlei Zhang, Lizhao Guan, Zhengquan Yu, Li Zhao

**Affiliations:** 1 Department of Biochemistry and Molecular Biology, School of Basic Medical Sciences, National Clinical Research Center for Cancer, Key Laboratory of Cancer Prevention and Therapy, Tianjin’s Clinical Research Center for Cancer, Tianjin Medical University Cancer Institute and Hospital, Tianjin Medical University, 22 Qixiangtai Road, Heping District, 300070 Tianjin, China; 2 Department of Nuclear Medicine, Tianjin Medical University General Hospital, 154 Anshan Road, Heping District, 300052 Tianjin, China; 3 State Key Laboratories for Agrobiotechnology, College of Biological Sciences, China Agricultural University, 2 Yuanmingyuan West Road, Haidian District, 100094 Beijing, China

**Keywords:** Cancer stem cells, Squamous cell carcinoma

## Abstract

Emerging evidence suggests that the cancer stem cells (CSCs) are key culprits of cancer metastasis and drug resistance. Understanding mechanisms regulating the critical oncogenic pathways and CSCs function could reveal new diagnostic and therapeutic strategies. We now report that *miR-22*, a miRNA critical for hair follicle stem/progenitor cell differentiation, promotes tumor initiation, progression, and metastasis by maintaining Wnt/β-catenin signaling and CSCs function. Mechanistically, we find that *miR-22* facilitates β-catenin stabilization through directly repressing citrullinase PAD2. Moreover, *miR-22* also relieves DKK1-mediated repression of Wnt/β-catenin signaling by targeting a FosB-DKK1 transcriptional axis. *miR-22* knockout mice showed attenuated Wnt/β-catenin activity and Lgr5^+^ CSCs penetrance, resulting in reduced occurrence, progression, and metastasis of chemically induced cutaneous squamous cell carcinoma (cSCC). Clinically, *miR-22* is abundantly expressed in human cSCC. Its expression is even further elevated in the CSCs proportion, which negatively correlates with PAD2 and FosB expression. Inhibition of *miR-22* markedly suppressed cSCC progression and increased chemotherapy sensitivity in vitro and in xenograft mice. Together, our results revealed a novel *miR-22*-WNT-CSCs regulatory mechanism in cSCC and highlight the important clinical application prospects of *miR-22*, a common target molecule for Wnt/β-catenin signaling and CSCs, for patient stratification and therapeutic intervention.

## Introduction

Due to its higher risk of recurrence and metastasis characteristics, cutaneous squamous cell carcinoma (cSCC) is responsible for the majority of non-melanoma skin cancers (NMSC) related deaths even it only accounts for 20% of the cases [1, 2]. Accumulating evidence suggests that metastasis and resistance to chemotherapy of cSCC are mainly attributed to the role of constitutive activated oncogenic pathways and CSCs [3–6]. For example, Latil et al. found that Lgr5-positive hair follicle stem cells (HFSCs) prefer to initiate cSCC with high metastatic potential when *KRas*^*G12D*^ expression and *p53* deletion were incorporated in a cell-type-specific manner [7]. In this context, the over-activated RAS signaling is the key premise of tumor formation and metastasis. Thus understanding key factors involved in modulating constitutive activated oncogenic pathways and CSCs function holds the promise to identify reliable biomarkers for diagnosis stratification as well as for therapeutic targeting.

Dysregulation of miRNAs has been reported in tumor initiation, progression, and response to therapy, thus are accepted as potential cancer biomarkers [8]. cSCC originates from either HFSCs or basal epidermal progenitors, where miRNA are playing pivotal roles during normal skin progenitor maintenance, malignant transformation, as well as CSCs function [9, 10]. Independent studies have shown that *miR-21* and miR-21* are highly expressed in CSCs of cSCCs and correlate with poor prognosis [11]. On the contrary, *miR-203* limits cell division in both early embryonic skin development and cSCC CSCs, so that its deficiency leads to the formation of poorly differentiated cSCC [12, 13]. The miRNA expression profiles during cSCC development and their potential therapeutic significance remains to be explored.

*MiR-22*, which has been reported to be dysregulated in various types of cancers, is involved in cancer cell proliferation, apoptosis, invasion, metastasis, and stemness [14–17]. In leukemia and breast cancer, *miR-22* promotes stem cell self-renewal and transformation by targeting the tumor suppressor TET2 so that its aberrant expression correlates with poor survival [18, 19]. During skin development, we found that *miR-22* is required for HFSC differentiation and hair follicle regression in vivo [20]. *MiR-22* level was also increased during HRas-induced malignant cSCC and exhibited 3.92 fold change in CSCs compared to adult stem cell [11]. These findings point to the potential function of *miR-22* in CSCs maintenance and cSCC development.

Here we found that *miR-22* expression is upregulated and positively correlated with cSCC severity. Functional studies unravel the role of *miR-22* in promoting cSCC initiation, progression, and metastasis in vitro and in vivo. Lineage tracing study showed that Lgr5^+^ CSCs contribute to the development of cSCC and metastasis, but abrogated in *miR-22*-deficient mice. Mechanism investigation and antagomir treatment results confirmed a critical *miR-22*-WNT-Lgr5^+^ CSCs regulatory axis in cSCC development. Our study highlights the important function of *miR-22* in cSCC tumor development and indicates its promising clinical significance as a diagnosis and therapeutic target.

## Results

### *miR-22* is upregulated in cSCC and correlated with cSCC progression and poor prognosis

**Fig. 1:**
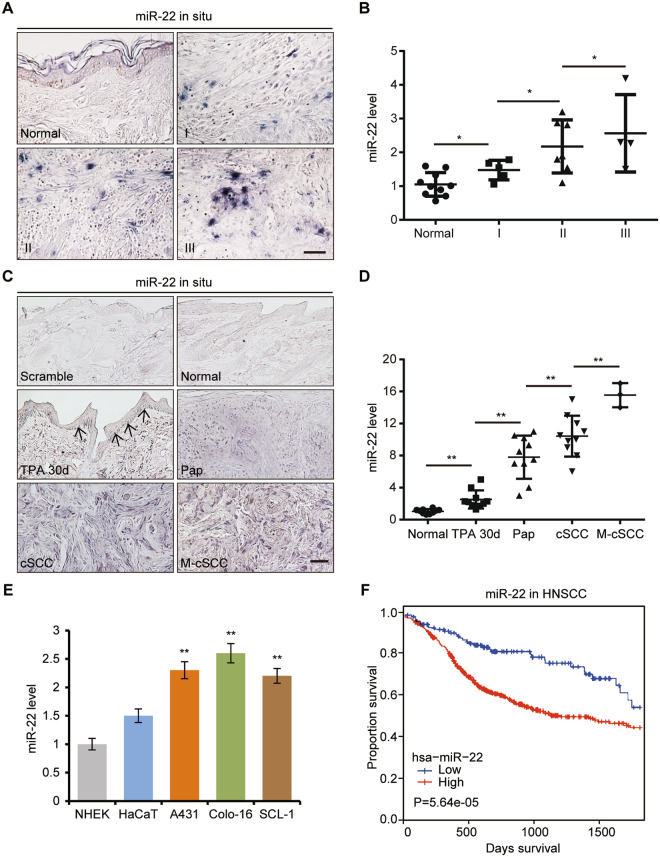
*miR-22* is highly expressed in cSCC and correlated with cancer progression and poor prognosis. **A** The expression level of *miR-22* in human normal skin (*n* = 17) and cSCC (grade I, *n* = 52; grade II, *n* = 34; grade III, *n* = 14) were checked by in situ hybridization. Scale bar, 50 µm. **B** The expression level of *miR-22* in human normal skin (*n* = 10) and cSCC (grade I, *n* = 5; grade II, *n* = 7; grade III, *n* = 4) were checked by qPCR. *p < 0.05. **C**, **D** In situ hybridizations and qPCR analysis of *miR-22* level in normal mouse skin (*n* = 10), TPA treated 30d skin (*n* = 10), papilloma (Pap, *n* = 10), cSCC (*n* = 10), and groin metastatic cSCC (M-cSCC, *n* = 3) induced by DMBA/TPA. Scale bar, 50 µm. **p < 0.01. **E** Expression levels of *miR-22* in HaCaT and cSCC cell lines (A431, Colo-16, and SCL-1) compared with NHEK cell lines were measured by qPCR. **p < 0.01. **F** miRNA transcriptome data from the TCGA database showed that HNSCC patients with higher *miR-22* expression levels show a trend towards decreased survival. A total of 522 HNSCC patients were included. There are 391 patients with high miR-22 expression levels and 131 patients with miR-22 expression levels.

Stem cells from hair follicle and epidermis could both contribute to cSCC development, we thus wonder whether *miR-22*, a critical regulator of HFSC differentiation, could be involved in cSCC occurrence. We conducted in situ hybridizations and qRT-PCR of *miR-22* on human cSCC tissue and DMBA/TPA induced mice cSCC tissues. Both results showed the ascending tendency of *miR-22* expression levels was positively correlated with human cSCC grading and malignant degree of mice cSCC (Fig. 1A–D and Supplementary Fig. 1a, b). Notably, the expression level of *miR-22* in metastatic carcinoma was significantly higher than that in primary carcinoma (Fig. 1C, D). Consistently, *miR-22* was more abundantly expressed in cSCC cell lines than in normal keratinocyte cell lines (Fig. 1E). In addition, we analyzed available head and neck squamous cell carcinoma (HNSCC) samples in the TCGA database and revealed that miR-22 was highly expressed in HNSCC compared to normal adjacent tissues, and *miR-22*^*high*^ tumors were associated with worse clinical outcomes than *miR-22*^*low*^ tumors, with regard to 5-year overall survival (Fig. 1F and Supplementary Fig. 1c, d). These findings indicate that *miR-22* could be playing an oncogenic role during cSCC tumorigenesis and metastasis.

### *miR-22* is critical for tumor cell migration and CSCs maintenance

**Fig. 2:**
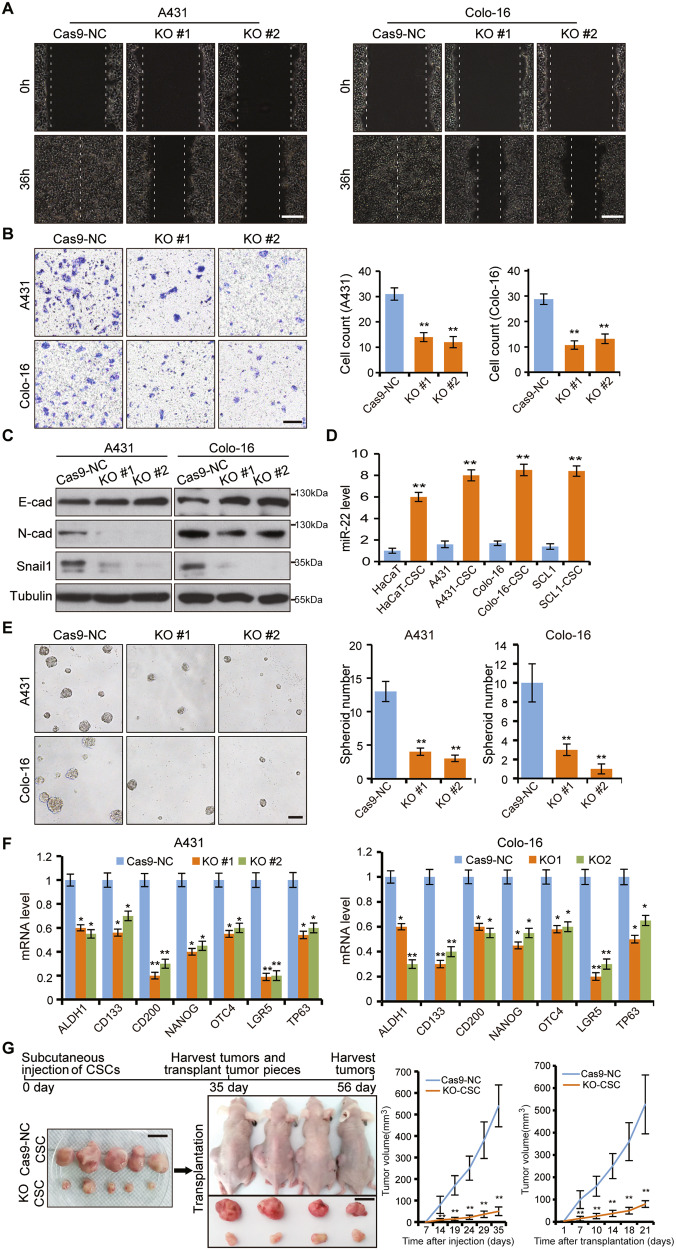
Loss of *miR-22* represses tumor metastasis and CSCs function. **A**, **B** *miR-22* was knocked out by CRISPR–Cas9 in A431 and Colo-16 cells. Cas9-NC indicated the negative control cell line. KO #1 and KO #2 indicated two miR-22 knockout cell lines by different gRNA. Cell migration was checked by cell scratch and transwell tests. The number of migrated cells per microscope field of view in transwell was counted. Scale bar, 200 µm. **C** Protein level of E-cad, N-cad, and Snail were determined by western blot (WB) in Cas9-NC and *miR-22* KO cells. **D** Expression levels of *miR-22* in HaCaT, A431, Colo-16, and SCL-1 compared with corresponding CSCs (spheroid-derived cells) were checked by qPCR. **E** Spheroid formation efficiency of Cas9-NC cells and *miR-22* KO cells were analyzed by suspension culture. The number of spheroid per microscope field of view was counted. Scale bar, 100 µm. **F** The mRNA level of stem cell-related markers was checked by qPCR. **G** CSCs from Cas9-NC cells and *miR-22* KO cells were injected subcutaneously in NOD/SCID mice (*n* = 5) and then the tumor pieces (~3 mm) from each group were inserted into the incision made in the dorsal skin of BALB/c nude mice (*n* = 4). The growth curves of tumors were generated and statistically compared. Scale bar, 1 cm. All the in vitro experiments were tested in three biological replicates. *p < 0.05, **p < 0.01.

To investigate whether miR-22 has a direct function during cSCC tumorigenesis, we manipulated miR-22 levels in cSCC cell lines (Supplementary Fig. 2a–c). Cell migration and epithelial–mesenchymal transition (EMT) activity were significantly reduced upon miR-22 deficiency (Fig. 2A–C). While miR-22 overexpression caused the opposite effect (Supplementary Fig. 2f–h). Additionally, we found that knockout of miR-22 also resulted in decreased expression of miR-22 host gene (MIR22HG) (Supplementary Fig. 2d, e). To rule out the effect of downregulation of MIR22HG expression on cell migration and spheroid formation efficiency, we repeated the cell scratch, transwell, and spheroid formation phenotype experiments with a conventional inhibitor of miR-22. These results showed that cell migration and spheroid formation efficiency were significantly inhibited by a miR-22 inhibitor, which was consistent with those of miR-22 KO cell lines (Supplementary Fig. 3a–e). At the same time, clone formation and CCK8 assays showed that miR-22 has no significant effect on cell proliferation in vitro (Supplementary Fig. 3f, g).

To determine whether miR-22 is similarly important in vivo, we established a xenograft model by injecting cSCC Colo-16 cells through mice tail veins. Colo-16 cells showed high migration potential with the majority landed on lungs within 6 weeks of injection, which were positive for cSCC cell marker K14. However, the incidence of lung metastasis was markedly decreased and K14^+^ cells were hardly detectable in a miR-22 knockout group (Supplementary Fig. 4a, b). Expression of Snail and N-cad were also decreased in miR-22-deficient tumors, which possibly explained the compromised metastatic capability (Supplementary Fig. 4b).

Considering the EMT promotion role together with the higher expression of *miR-22* in metastatic carcinoma compared to on-site carcinoma, we reasoned that *miR-22* promotes cSCC cell metastasis through regulating CSCs function. As predicted, expression of *miR-22* was higher in cell spheroids with CSCs signatures enriched by suspension culture compared with adherent cultured cells (Fig. 2D and Supplementary Fig. 4c). Furthermore, the spheroid formation efficiency was decreased by knocking out *miR-22* (Fig. 2E, F). In contrast, overexpression of *miR-22* promoted CSCs expansion and formed bigger size spheroids (Supplementary Fig. 4d).

An essential property of CSCs is their ability to self-renew and reconstitute tumor heterogeneity during serial transfers into new recipient mice [21]. To further explore the role of *miR-22* on CSCs in vivo, CSCs (spheroid-derived cells) from *miR-22* knocked-out cell lines were injected subcutaneously into NOD/SCID mice in parallel with control cells, thereafter tumors formed were transferred into new recipient mice (BALB/c nude mice). The results showed that *miR-22* knocked-out CSCs gave rise to much smaller tumors than control CSCs in both the primary and passaged tumors. Moreover, visual inspection indicated that the control CSCs-derived tumors were better vascularized (Fig. 2G). Collectively, the ability of CSCs to form multi-lineage tumors was abrogated after *miR-22* deficiency, which could not recover even after tumor passaging. These findings confirmed the necessity of *miR-22* in CSCs maintenance and high *miR-22* expression may be an important feature of CSCs function.

### *miR-22* is essential for CSCs to initiate and sustain tumor growth in vivo

**Fig. 3:**
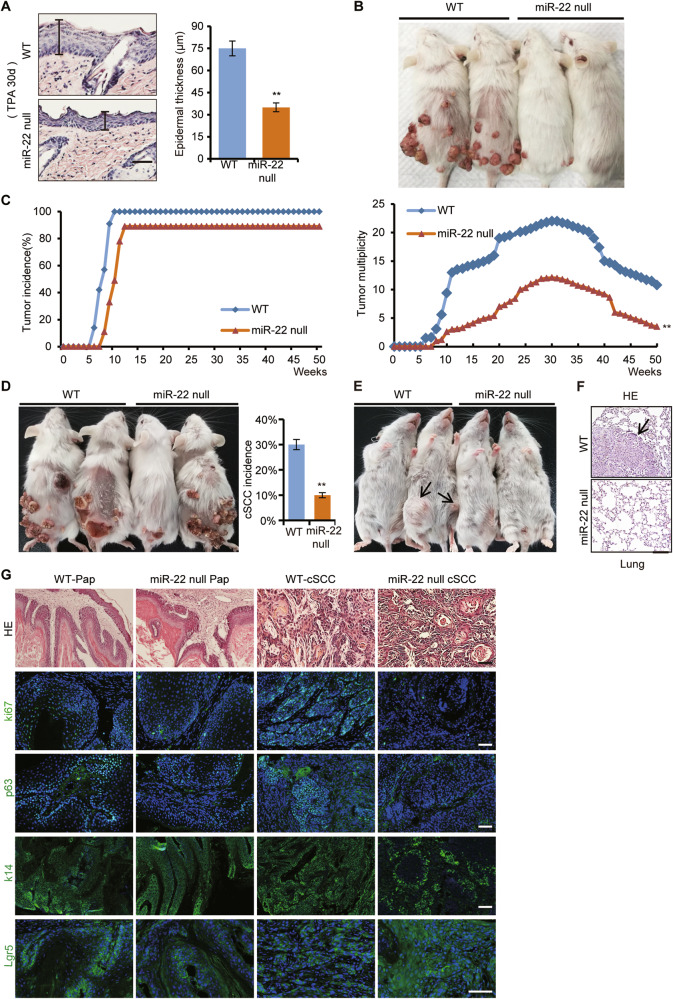
*miR-22* is essential for CSCs to initiate and sustain tumor growth in vivo. **A** The epidermis thickness of WT (*n* = 5) and *miR-22* null mice (*n* = 5) were checked after a DMBA initiation and 30 d TPA promotion treatment. Scale bar, 50 µm. **B** Papilloma occurred on WT (*n* = 10) and *miR-22* null mice (*n* = 10) dorsal skin after continuous TPA treatment. **C** Tumor incidence and multiplicity of WT (*n* = 10) and *miR-22* null mice (*n* = 10) were shown. **p < 0.01. **D** cSCC occurred on WT (*n* = 10) and *miR-22* null mice (*n* = 4) dorsal skin and the incidence were counted. **E**, **F** Inguinal and lung metastases in WT (*n* = 3) and *miR-22* null mice. The arrows point to metastatic cancer. Scale bar, 50 µm. **G** Haematoxylin and eosin (HE) and immunofluorescence (IF) staining for ki67, p63, K14, and Lgr5 in papilloma and cSCC from WT (*n* = 3) and *miR-22* null mice (*n* = 3). Scale bar, 50 µm.

Based on the similar expression and in vitro function of *miR-22* between mice and human, we further explored *miR-22* function in vivo, by combining the genetic knockout mouse and DMBA/TPA induced cSCC model [22]. As shown in Fig. 3A–C, the epidermis hyperplasia was severely inhibited and tumor initiation was significantly delayed in *miR-22* null mice compared to their WT littermates. There were fewer and smaller papillomas in *miR-22* null mice. Consequently, the incidence of both orthotopic malignant cSCC and inguinal and lung metastases was markedly decreased. Histologically, the cSCC from *miR-22* null mice was less aggressive according to the SCC Broders’ pathologic classification (Fig. 3D–G) [23, 24]. These results showed that *miR-22* deficiency suppressed cSCC initiation, progression, and metastasis.

As we have mentioned, CSCs possess the capacity to reconstitute tumor hierarchy and cellular heterogeneity, which are thus also called tumor-initiating cells (TICs) [25]. Through immunostaining, we found that the hyper-proliferation ability of tumor cells was inhibited in both papilloma and cSCC tissues from *miR-22* null mice compared to WT mice (Fig. 3G). Additionally, the abundance of common cSCC CSCs, represented by p63, K14, and Lgr5 signals, was severely reduced in *miR-22* null mice (Fig. 3G). Therefore, loss of *miR-22* hindered the tumorigenesis probably by affecting the activity of CSCs.

### Lgr5^+^ stem cells contribute to cSCC development and metastasis, but abrogated in *miR-22*-deficient mice

**Fig. 4:**
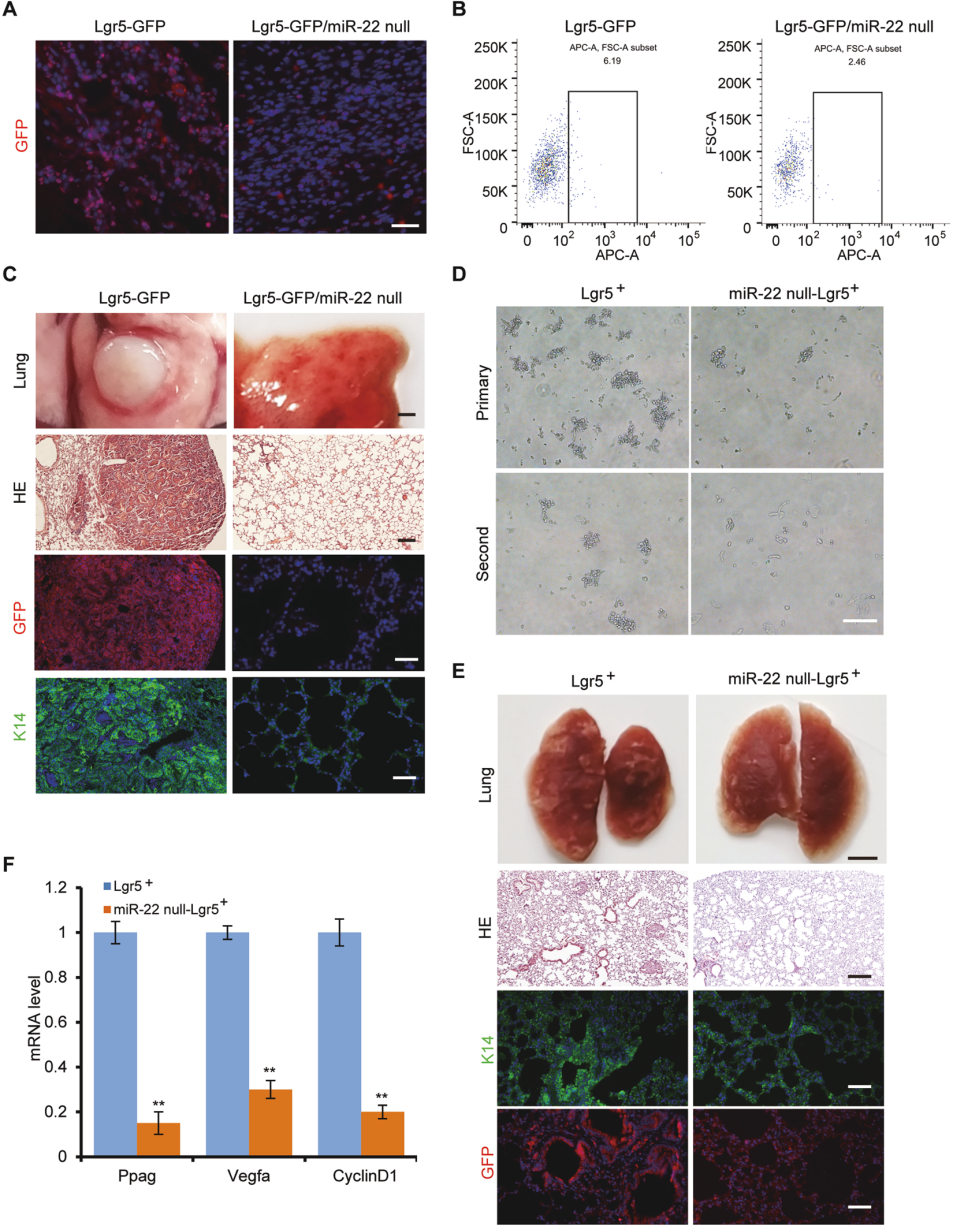
*miR-22* deficiency prohibited the participation of Lgr5^+^ stem cells into cSCC and metastasis. **A** IF analysis for GFP in cSCC of *Lgr5-GFP* (*n* = 3) and *Lgr5-GFP/miR-22 null* mice (*n* = 3). Scale bar, 50 µm. **B** The proportion of Lgr5-positive cells (Lgr5^+^) in cSCC of *Lgr5-GFP* mice (*n* = 3) and *Lgr5-GFP/miR-22 null* mice (*n* = 3) were analyzed by flow cytometry using an anti-Lgr5 antibody. **C** HE and IF analysis for GFP and K14 in lung metastasis of *Lgr5-GFP* (*n* = 3) and *Lgr5-GFP/miR-22 null* mice (*n* = 3). The scale bar of lung tissue, HE, GFP, and K14 images were 1250, 200, 100, and 100 µm, respectively. **D** The Lgr5^+^ cells were subjected to non-attached culture after sorting, and the capability of sphere-forming was compared after continuous cell passaging. Scale bar, 100 µm, *n* = 3 biological replicates. **E** HE and IF analysis for GFP and K14 in lung metastasized cancer tissue of *NOD/SCID* mice, which were injected with Lgr5^+^ cells through tail veins. The scale bar of lung tissue, HE, GFP, and K14 images were 1250, 200, 100, and 100 µm respectively. *n* = 3 biological replicates. **F** mRNA level of Wnt pathway downstream target genes was checked by qRT-PCR in Lgr5^+^ and *miR-22* null- Lgr5^+^ cells. **p < 0.01. *n* = 3 biological replicates.

Lgr5^+^ cells, as an important HFSCs population, give rise to epidermis cells during skin development and have a high potential to function as CSCs once KRas^G12D^ mutation is incorporated [7]. But under DMBA/TPA induced cSCC model, whether the Lgr5^+^ cells are playing the critical role is not clear. To have a global view of the trace and contribution of Lgr5^+^ cells in DMBA/TPA induced cSCC model, *Lgr5-EGFP-IRES-CreERT2 (Lgr5-GFP)* mice were subjected to DMBA/TPA induction. Interestingly, incorporation of GFP positive cells in cSCC tissues was markedly decreased once *miR-22* was deleted in *Lgr5-GFP* mice (Fig. 4A). Quantification by FACS analysis using anti-Lgr5 antibody further confirmed that the number of originated Lgr5^+^ cells was also reduced (Fig. 4B). Consistently, seldom metastasis or K14^+^ cells were observed on the lung tissues from *Lgr5-GFP/miR-22 null* double mutant mice (Fig. 4C). What really drew our attention was that nearly all metastasized cells were GFP positive, which suggested their Lgr5^+^ cell origin. These lineage tracing results showed that Lgr5^+^ cells contribute to tumor formation and metastasis, which processes were remarkably abrogated by *miR-22* deficiency.

To further evaluate the stem cell property of Lgr5^+^ cells from induced tumor tissues as well as the function of *miR-22* within these cells, we analyzed their self-renewal and in vitro expansion ability. The anchorage-independent proliferating capacity of *miR-22 null*-Lgr5^+^ cells sorted by the anti-Lgr5 antibody was severely reduced (Fig. 4D). Furthermore, the pulmonary metastasis ability of *miR-22 null*-Lgr5^+^ cells was also severely impaired (Fig. 4E). Altogether, these data suggested that Lgr5^+^ cells could be functioning as seeding cells during cSCC development and metastasis and the cell maintenance depends very much on the presence of *miR-22*.

### *miR-22* maintains active Wnt/β-catenin signaling in cSCC

**Fig. 5:**
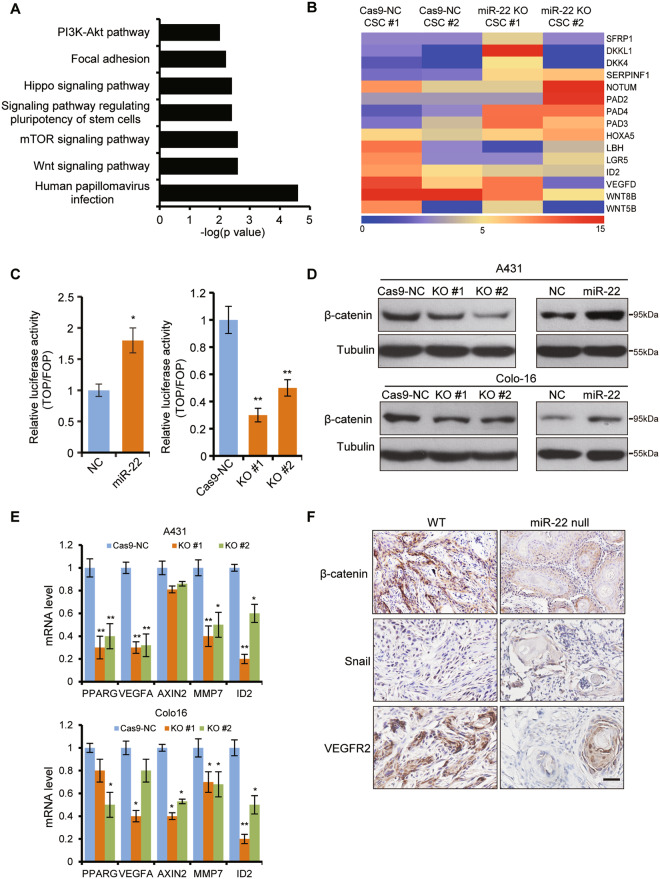
Wnt/β-catenin signaling was abrogated in *miR-22* knockout cells and tissues. **A** RNAseq of Cas9-NC (*n* = 2) and *miR-22* KO (*n* = 2) cells spheroids was performed and KEGG pathway enrichment of the downregulated genes was analyzed. **B** Heatmap for Wnt/β-catenin pathway inhibitors (SFRP1, DKKL1, DKK4, SERPINF1, NOTUM, PAD2, PAD4, PAD3, HOXA5) and effectors (LBH, LGR5, ID2, VEGFD, WNT8B, WNT5B). This heatmap was generated and analyzed based on the fold change of the differentially expressed genes between miR-22 knockout and wild-type cSCC cell spheroids. **C**, **D** TOPflash reporter activity, and β-catenin protein level was determined in *miR-22* overexpressed cells (*miR-22*) and its negative control cells (NC), Cas9-NC cells, and *miR-22* KO cells. *n* = 3 biological replicates. *p < 0.05, **p < 0.01. **E** The mRNA level of Wnt/β-catenin related downstream factors were analyzed by qPCR in Cas9-NC cells and *miR-22* KO cells. *p < 0.05, **p < 0.01. *n* = 3 biological replicates. **F** Immunohistochemistry (IHC) analysis for β-catenin, Snail, and VEGFR2 in cSCC from WT (*n* = 3) and *miR-22* null mice (*n* = 3). Scale bar, 50 µm.

To uncover the molecular mechanism through which *miR-22* regulates the CSCs population and promotes cSCC development, transcriptome comparisons were performed between *miR-22* knockout and wild-type cSCC cell spheroids. KEGG pathway analysis of the downregulated genes indicated that the signaling pathway regulating pluripotency of stem cells, Wnt/β-catenin signaling pathway, and human papillomavirus infection pathway were prominent pathways enriched (Fig. 5A). Human papillomavirus infection is a key cause of cSCC development. Additionally, there were 370 common genes between the upregulated genes of our transcriptome data and the downregulated genes with GSE2503 array data from the GEO database which detected the gene expression difference of human cSCC compared to normal skin. There were 315 common genes between the downregulated genes of our transcriptome data and the upregulated genes with GSE2503 array data (Supplementary Fig. 5a–c). Thus the transcriptome data further confirmed that *miR-22* was an oncogene in cSCC development and CSCs maintenance.

Wnt/β-catenin signaling was identified to be the most significantly enriched pathway in cSCC [26], which led us to explore the underlying mechanism. Interestingly, we found various Wnt/β-catenin pathway inhibitors were upregulated, while related effectors were downregulated in *miR-22* knockout spheroids (Fig. 5B). TOPflash reporter activity was dramatically repressed in *miR-22* knocked-out but elevated in overexpressed cells (Fig. 5C). The protein levels of β-catenin showed a similarly positive correlation with *miR-22* abundance in different cancer cells and cancer stem cells (Fig. 5D and Supplementary Fig. 5d). Functionally, the expression levels of related downstream factors were significantly decreased in *miR-22* knockout cells (Fig. 5E). The findings indicated that the Wnt/β-catenin pathway was indeed inhibited by knocking out *miR-22*.

Based on these in vitro analyses, we went back to the transgenic mouse models to examine the in vivo correlation between the Wnt/β-catenin pathway and *miR-22*. β-catenin was highly expressed and mostly localized in the nuclei of induced cancer tissues, which corresponded with increased metastasis potential and intense Snail signal (Fig. 5F). VEGFR2, as an effector of VEGF signaling and one of the most active responders to the Wnt/β-catenin pathway, also showed extremely faint levels in *miR-22* null tissues (Fig. 5F). Similarly, we detected a marked reduction of Wnt/β-catenin signaling effectors in *miR-22* null-Lgr5^+^ cells (Fig. 4F). These results indicated that *miR-22* could regulate cSCC formation and CSCs function through promoting Wnt/β-catenin signaling.

### *miR-22* directly targets *FOSB* and *PAD2* to promote cSCC development

**Fig. 6:**
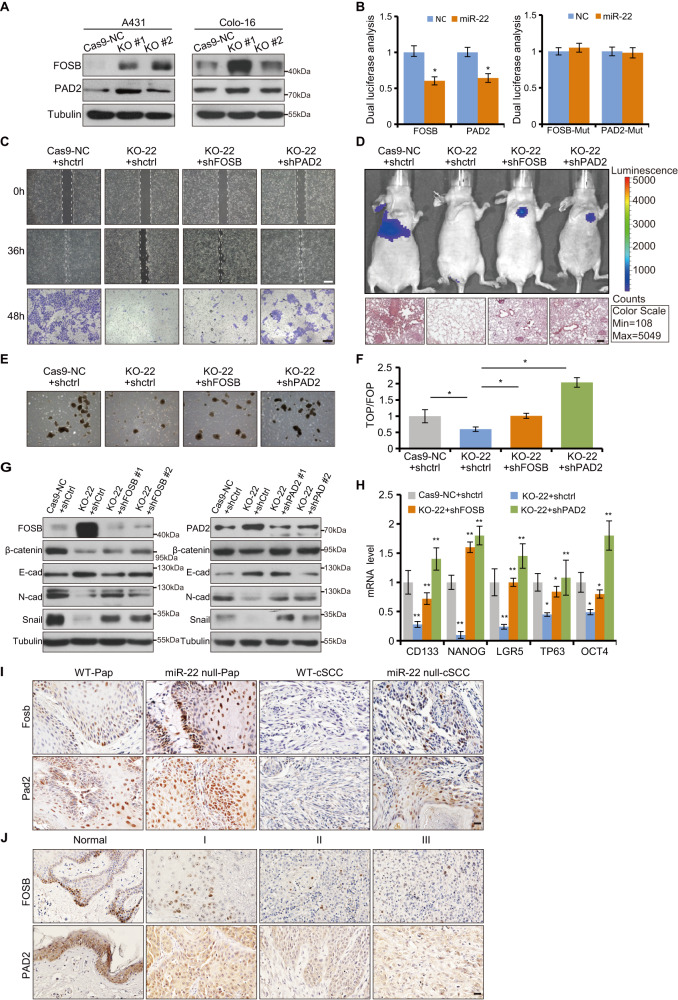
*FOSB* and *PAD2* were direct and functional targets *of miR-22* in cSCC. **A** FOSB and PAD2 protein levels were detected in Cas9-NC cells and *miR-22* KO cells by western blot. **B** Luciferase activity of *FOSB-3*′*UTRs*, *PAD2-3*′*UTRs* reporter constructs, and their *miR-22* binding site mutated constructs were analyzed in *miR-22* overexpressed cells (*miR-22*) and its negative control cells (NC). **C** Knockdown of *FOSB* or *PAD2* rescued the migration deficiency caused by *miR-22* knockout in cSCC cell lines respectively. Cas9-NC + shctrl, KO-22 + shctrl, KO-22 + shFOSB and KO-22 + shPAD2 were indicated Cas9-NC A431 cell line transfected with control shRNA, *miR-22* knockout cell line transfected with shctrl, FOSB shRNA, and PAD2 shRNA respectively. Scale bar, 200 µm. **D** Cas9-NC + shctrl, KO-22 + shctrl, KO-22 + shFOSB, and KO-22 + shPAD2 Colo-16 cells were injected into BALB/c nude mice (*n* = 5) through tail veins respectively and visualized through Caliper IVIS Spectrum System. One month after injection, histological analysis of lungs from corresponding nude mice was conducted through HE staining. Scale bar, 50 µm. **E**, **F** Spheroid formation efficiency and TOPflash reporter activity were checked in Cas9-NC + shctrl, KO-22 + shctrl, KO-22 + shFOSB and KO-22 + shPAD2 A431 cells. **G** Protein level of FOSB, PAD2, β-catenin, E-cadherin, N-cadherin, and Snail were checked in corresponding A431 cells by WB. **H** The mRNA level of CSC markers were rescued by knockdown *FOSB* or *PAD2* in *miR-22* knockout A431 cell line, respectively. **I**, **J** IHC analysis for FOSB and PAD2 in Pap and cSCC of WT and *miR-22* null mice, and human cSCC with different pathological grade. Scale bar, 50 µm. All the in vitro experiments were tested in three biological replicates. *p < 0.05, **p < 0.01.

To identify *miR-22* targets that are responsible for its tumorigenic effects, we compared the downregulated genes in human cSCC transcriptomes from GSE2503 array data and upregulated genes in our transcriptome data in combination with software predicted *miR-22* target genes. Roughly, we identified *FOSB*, *PAD2*, and the other 8 factors as putative *miR-22* targets. QRT-PCR and western blot analysis confirmed that *FOSB*, *PAD2*, and *HOXA5* were the most upregulated three genes in *miR-22* knockout cells and Pap tissues (Fig. 6A and Supplementary Fig. 6a–d). Luciferase activity of *FOSB* and *PAD2* 3′UTRs were significantly repressed by *miR-22* overexpression, which was, however, abolished when the sites were mutated (Fig. 6B). Nevertheless, *HOXA5* 3′UTR activity was not affected by *miR-22* overexpression (Supplementary Fig. 6e). Furthermore, we found that FOSB and PAD2 function as cell migration suppressors in vitro which are opposite to *miR-22* function (Supplementary Fig. 6f–i). Therefore, the screenings gradually narrowed *miR-22* targets down to *FOSB* and *PAD2* in our model.

Indeed, knockdown of *FOSB* or *PAD2* rescued the cell migration, lung metastasis, and spheroid formation efficiency defect caused by *miR-22* knockout in cSCC cell lines respectively (Fig. 6C–E and Supplementary Fig. 7a–d). Then the TOPflash activity results showed that the observed functional recovery of cSCC cell behaviors was related to Wnt/β-catenin signaling activity (Fig. 6F). Molecularly, the expression of β-catenin, EMT markers, and CSCs markers showed effective but differential rally (Fig. 6G, H). Collectively, tumorigenesis and CSCs function suppression caused by *miR-22* deficiency could be due to repression release of *FOSB* or *PAD2* and concomitant Wnt/β-catenin signaling blockage.

More importantly, histological analysis showed that expression of FOSB and PAD2 were significantly downregulated as the pathological grade of human and mouse cSCC progresses, negatively correlated with *miR-22* (Figs. 1A–D and 6I, J). Additionally, we also found that *FOSB* and *PAD2* levels were downregulated as the pathological grade of human cSCC progresses within GSE2503 array data (Supplementary Fig. 8a, b). Collectively, *miR-22* seems to exert the oncomiR function mostly through repressing tumor suppressors *FOSB* and *PAD2* in cSCC.

### FOSB and PAD2 mediates regulation of Wnt/β-catenin signaling by *miR-22* via modulating DKK1 and β-catenin

**Fig. 7:**
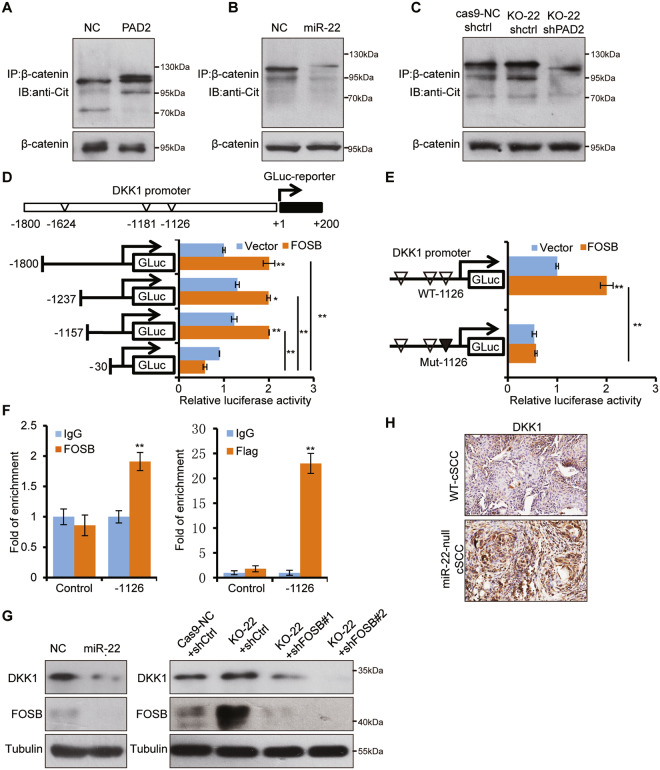
PAD2 and FOSB mediates regulation of Wnt/β-catenin signaling by miR-22 through promoting β-catenin degradation and DKK1 transcriptional activation. **A**–**C** β-catenin was immunoprecipitated and WB with antibodies against citrulline and β-catenin from indicated cell lines separately. **D** Potential FOSB-binding sites within *DKK1* promoter were mapped. HEK293T cells were co-transfected with different GLUC constructs of DKK1 promoter and FOSB overexpressed plasmid or control plasmid to perform GLUC activity assays. **E** FOSB-binding site (−1126) within DKK1 promoter were mutated and subjected to GLUC activity assay. **F** ChIP experiments were performed using anti-FLAG in A431 cells with stable overexpression of FLAG-tagged FOSB or anti-FOSB antibody in normal A431 cells, and followed by qRT-PCR with primers across the regions containing potential FOSB-binding site (−1126) in *DKK1* promoter. Control indicated a negative control promoter sequence without an FOSB-binding site. **G** Protein level of DKK1 and PAD2 were checked in indicated cells by WB. **H** IHC analysis for DKK1 in cSCC of WT and miR-22 null mice. All the in vitro experiments were tested in three biological replicates. *p < 0.05, **p < 0.01.

To understand how Wnt/β-catenin signaling was modulated by *miR-22*, we further explored the underlying molecular mechanisms. PAD2 has been found to promote β-catenin degradation through citrullination modification in colorectal cancer [27]. Similarly, PAD2 overexpression led to the significant increase of global protein citrullination level, particularly that of β-catenin, but *miR-22* overexpression showed the opposite effects (Fig. 7A, B and Supplementary Fig. 9a). On the contrary, β-catenin citrullination was elevated in *miR-22* knockout cells, which partially explained the reduced β-catenin. Significantly, further PAD2 knockdown brought the citrullination to a hardly detectable level (Fig. 7C). These results suggested that *miR-22* promotes β-catenin stabilization probably through inhibiting PAD2-mediated citrullination.

With regard to FOSB, it normally functions as a transcriptional activator, so we hypothesized that FOSB may inhibit the Wnt/β-catenin pathway by transcriptionally activating certain inhibitors. By screening Wnt/β-catenin related inhibitors and our transcriptome data, factors represented by *DKK1*, *DKK3* drew our attention due to their elevation in *miR-22* knockout tumors. qPCR quantification showed that only *DKK1* was consistently downregulated in different cell lines after FOSB knockdown (Supplementary Fig. 9b, c). Moreover, the protein level of DKK1 altered in a synchronizing way with FOSB knockdown or overexpression (Supplementary Fig. 9d).

To determine whether *DKK1* is transcriptionally controlled by FOSB, we constructed a series of GLUC reporters containing full-length or truncated *DKK*1 gene promoter regions containing predicted FOSB-binding sites (Fig. 7D). The GLUC reporters results confirmed that −1126 site of *DKK1* promoter was the FOSB-mediated transcriptional activation site (Fig. 7D, E). Further endogenous and exogenous CHIP assays verified that FOSB activates *DKK1* promoter through direct binding to the region around −1126 sites (Fig. 7F). Taken together, these results demonstrated that FOSB physically binds to the *DKK1* promoter and represses its transcription.

To fit FOSB-mediated *DKK1* transcriptional activation into *miR-22* regulation of Wnt/β-catenin pathway activity, we checked whether the DKK1 level was truly different within *miR-22* varied groups. Interestingly, DKK1 negatively responded to *miR-22* overexpression and knockout at both mRNA and protein levels (Fig. 7G and Supplementary Fig. 9e). Further knockdown of FOSB in *miR-22* knockout cells failed to bring DKK1 up, thus confirming the hinge role of FOSB between *miR-22* and DKK1 (Fig. 7G). In vivo, we also found the most convincing evidence that DKK1 staining was markedly elevated in *miR-22* null cSCC tissue (Fig. 7H). Therefore*miR-22* maintains Wnt/β-catenin pathway activity both through repressing PAD2-mediated β-catenin degradation and FOSB-mediated DKK1 activation.

### *miR-22* antagonism could be a therapeutic strategy for cSCC

**Fig. 8:**
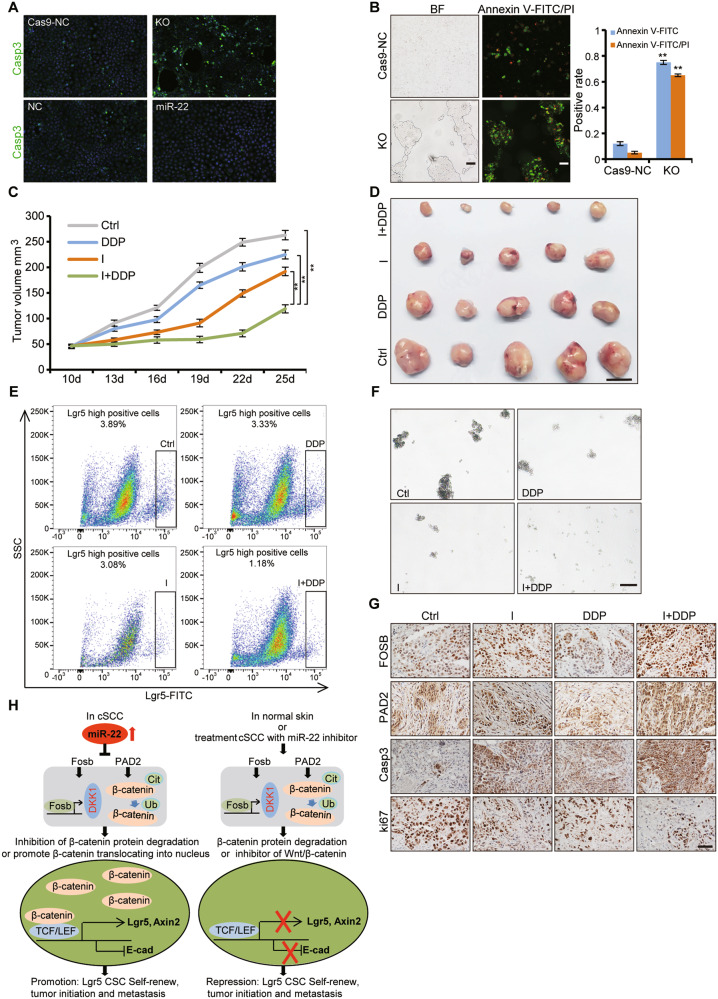
Inhibition of miR-22 promotes the sensitivity of cSCC cells to cisplatin. **A** Apoptotic cells were analyzed by IF for cleaved caspase-3 (Casp3) in Cas9-NC, *miR-22* KO, NC, and *miR-22* cells. Scale bar, 50μm. *n* = 3 biological replicates. **B** After cisplatin treatment, apoptotic and necrotic cells were analyzed by IF for Annexin-FITC and PI in Cas9-NC and *miR-22* KO cells. Scale bar, 50 μm. *n* = 3 biological replicates. **p< 0.01. **C** Growth curve of subcutaneous tumor in BALB/c nude mice after treatment with *miR-22* antagomir (antagonist) (I), cisplatin (DDP), and *miR-22* antagomir plus cisplatin (I + DDP) was shown. Ctrl indicated the untreated group. **p < 0.01. *n* = 5 biological replicates. **D** The mice were sacrificed at the end of the treatment experiment and images were taken along with the dissected tumors from five representative mice are shown. Scale bar, 1 cm. *n* = 5 biological replicates. **E** The proportion of Lgr5**-**positive cells in tumors was analyzed by flow cytometric analysis after treatment. *n* = 5 biological replicates. **F** The capability of sphere-forming among primary tumor cells with different treatment were analyzed. Scale bar, 100 μm. *n* = 5 biological replicates. **G** IHC analysis for FOSB, PAD2, cleaved caspase-3 and ki67 in tumor tissues after different treatment. Scale bar, 50 μm. *n* = 5 biological replicates. **H** Scheme of function and regulation mechanism of *miR-22* during the tumorigenesis of cSCC. During the initiation and development of cSCC, *miR-22* is upregulated and represses expression of FOSB and PAD2, which sequentially caused the downregulation of DKK1 and degradation-release of β-catenin. The accumulative effect is represented with activated Wnt/β-catenin signaling and consequent Lgr5^+^ stem cells expansion so as to tumorigenesis. However, in the normal skin or cSCC treated with *miR-22* antagonist, the above regulatory cascades are kept at an innocent state so that Lgr5^+^ stem cells stay quiescent and tumor formation is not initiated.

Constitutive activation of oncogenic pathways and increased abundance of CSCs take most of the responsibility for cancer metastasis and drug resistance. Based on the function of *miR-22* on WNT signaling and CSCs, we wonder whether targeting *miR-22* could achieve the therapeutic effect of targeting WNT signaling and CSCs simultaneously. We actually observed that *miR-22* knockout promoted cSCC cell apoptosis and the apoptotic and necrotic cells increased more significantly when combined with cisplatin treatment in vitro (Fig. 8A, B). To further examine the therapeutic function of *miR-22* antagonist in vivo, we performed intratumoral injection of *miR-22* antagonist simultaneously with an intraperitoneal injection of cisplatin on subcutaneously formed cSCC. Strikingly, combined therapy inhibited tumor growth much more effectively than either *miR-22* antagonist or cisplatin single therapy (Fig. 8C, D). We then analyzed the tumor cellular composition from different therapy groups by FACS, and found Lgr5^+^ cells were reduced according to the tumor volumes (Fig. 8E). The spheroid formation efficiency from primary tumor cells was also dramatically compromised after subjection to *miR-22* antagonist treatment (Fig. 8F). The therapy results indicate that *miR-22* antagonist may effectively repress cSCC development and progression through interfering Lgr5^+^ CSCs function, which further supports the indispensable role of *miR-22* in CSCs maintenance.

Under these circumstances, we were curious whether the Wnt/β-catenin pathway was affected by *miR-22* antagonist treatment in a similar manner as the induced tumor model. The expression of WNT signaling-related effectors was significantly downregulated in the tumor treated with *miR-22* inhibitor or combined with cisplatin compared to related control (Supplementary Fig. 10). Cell proliferation was reduced and apoptosis was elevated simultaneously with *miR-22* target genes FOSB, PAD2 (Fig. 8G). These findings not only indicated that specific targeting of *miR-22* may be an effective treatment for cSCC, especially for metastatic malignancies but also verified the functioning mechanism of *miR-22* in cSCC development.

## Discussion

In the current study, we found that *miR-22* expression is upregulated and positively correlated with cSCC severity. Its expression pattern emphasizes the potential that *miR-22* could act as biomarkers for the diagnosis and prognosis of clinical cSCC [11, 28–32]. As an oncomiR, *miR-22* plays an indispensable role in cancer initiation, progression, and metastasis. Lineage tracing study showed that Lgr5^+^ CSCs contribute to the development and metastasis of cSCC which are significantly compromised upon *miR-22* deficiency. Further mechanism exploration revealed a novel *miR-22*-WNT-CSCs regulatory mechanism in cSCC development and highlight the important clinical application prospects of *miR-22*, a common target molecule for WNT signaling and CSCs, for patient stratification and therapeutic intervention (Fig. 8H).

Lgr5^+^ HFSCs give rise to epidermis cells during skin development and have a high potential to function as CSCs once oncogenic gene mutations and Ras signaling is constitutive activated [7, 33]. In the DMBA/TPA induced cSCC mouse model, we did detect that Lgr5^+^ cells also contribute to the formation of primary and lung metastatic cSCC. Overall, different independent genetic models all point to the existence and powerful function of this cell population, therapy targeting Lgr5^+^ cells may become a promising strategy. According to our expression analysis, *miR-22* is mainly expressed in the outer root sheath (ORS) of hair follicles where locate the majority of Lgr5^+^ cells [20]. It’s reasonable to ask whether the level of *miR-22* could affect the content and behavior of the Lgr5^+^ cells. With the absence of *miR-22*, less Lgr5^+^ cells were maintained so that cSCC initiation was delayed and metastasis was relieved. Additionally, we also found that the self-renew of K14^+^ and p63^+^ CSCs was inhibited in both Pap and cSCC tissues of *miR-22* null mice. Therefore, *miR-22* exerts its oncomiR function mostly through interfering with CSCs abundance and the function to tumorigenesis and metastasis.

Lgr5^+^ cells have been found to be under regulation by Wnt/β-catenin signaling during normal organogenesis as well as tumorigenesis [34, 35]. Furthermore, cutaneous CSCs maintenance is dependent on Wnt/β-catenin signaling, and ablation of the β-catenin gene results in the loss of CD34^+^ CSCs and complete tumor regression [36]. In the current study, the downregulated activity of Wnt/β-catenin signaling was actually the most significantly affected cascade accompanying the loss of Lgr5^+^ cells in *miR-22*-deficient models, which thus confirmed the critical regulating role on these cell population. Mechanism explores found that *miR-22* promotes Wnt/β-catenin signaling activity via targeting *PAD2* and *FOSB* with consequent β-catenin stabilization and DKK1 reduction. Therefore the Wnt/β-catenin signaling which is one of the most attractive targets signaling for cSCC therapy is under delicate control by *miR-22* at different levels [37]. This means that targeting *miR-22* has become a promising new strategy for targeting Wnt/β-catenin signaling in cSCC. Indeed, we observed markedly suppressed WNT signaling in induced cSCC tissues of *miR-22*-deficient mice and subcutaneous tumor after treatment with *miR-22* antagonist. The more attractive advantage of *miR-22* as a target for targeting WNT signaling is that it could effectively block WNT/β-catenin signaling in cSCC or even with APC or CTNNB1 mutations by promoting degradation of citrullinated β-catenin [27, 38, 39]. Although our current therapy experiments were based on the cSCC model in immune-deficient mice, its therapeutic effect is similar to the phenotype of DMBA/TPA induced *miR-22* null mice. Apparently, drug packaging and delivering strategies with elevated biological stability and targeting efficacy for *miR-22* inhibitors deserve further development, and Nano-conjugated medicine could be an option.

One thing that must be paid attention to is that the pathological function of miR-22 may depend on the context of different cancer types. Consistent with our results, miR-22 activated WNT signaling in glioblastoma by targeting Wnt inhibitor SFRP2 and PCDH15 [40]. But in colon cancer, the WNT signaling was inhibited by miR-22 through targeting BCL9L [17]. The abundance of miR-22 in these cancers and the level of related target transcripts, even the competition among these targets may impact the performance of miR-22.

We also realized that certain functional and regulatory mechanisms are skin tumor-specific. FOSB functions downstream of Ras/MAPK signaling as a tumor promoter most of the time. But in our study, FOSB shows suppressed expression in cSCC samples from clinical specimens to cell lines, and to animal tissues, partially due to highly expressed *miR-22*. The main biological significance seems to keep DKK1 at a pretty low level so that Wnt/β-catenin could get activated and maintain CSCs activity. Consistently, *FOSB* and *PAD2* expression are downregulated as the pathological grade of human cSCC progresses as shown in GSE2503 array data. In terms of therapeutic strategies based on inhibitors targeting Ras/MAPK pathway may need careful evaluation when applied to cSCC treatment.

Overall, our data uncover a novel *miR-22*-WNT-CSCs regulatory mechanism in cSCC and highlight the important clinical application prospects of *miR-22*, a common target molecule for targeting WNT signaling and CSCs, for patient stratification and therapeutic intervention.

## Materials and methods

### Ethics

We got patient cSCC tissue RNAs (Normal skin, *n* = 10; grade I cSCC, *n* = 5; grade II cSCC, *n* = 7; grade III cSCC, *n* = 4) from the tissue bank of Tianjin cancer institute and hospital (Tianjin, China) under protocols approved by the Ethics Committee of Tianjin Medical University. All mouse experiment procedures and protocols were evaluated and authorized by the Regulations of Tianjin Laboratory Animal Management and strictly followed the guidelines under the Institutional Animal Care and Use Committee of Tianjin Medical University (Accreditation number: SYXK (Tianjin) 2016-00012).

### Mice

*MiR-22* null mice (Stock No: 018155) and Lgr5-EGFP-IRES-CreER (Stock No: 008875) mice were kindly gifted by Professor Zhengquan Yu of China Agricultural University. Their genetic background was replaced with FVB strain by backcrossing at least ten generations onto FVB wild-type mice. BALB/c nude mice and NOD/SCID mice were purchased from Beijing Vital River Laboratory Animal Technology Co., Ltd.

### Skin cancer tissue chip

Paraffin-embedded skin cancer tissue chips were purchased from Xi’an Alenabio Biotechnology Co., Ltd and Shanghai Outdo Biotech Co., Itd. A total of 100 cases of skin squamous cell carcinoma with different grade (60 from men and 40 from women) and 17 cases of normal skin tissues in these chips. Approximately 95% of the patients were more than 50 years of age. Tumors were classified according to the SCC Broders’ pathologic classification [23, 24]: grade I (well-differentiated) with 75–100% differentiated cells, grade II (moderately differentiated) with 50–75% differentiated cells, and grades III and IV (poorly differentiated) with 0–50% differentiated cells.

### Analysis of The Cancer Genome Atlas (TCGA) data

For human HNSCC patient data, level 3 miRNAseq data and clinical data were downloaded via DataPortal at The Cancer Genome Atlas (TCGA: http://cancergenome.nih.gov). The normalized reads per million quantification for miR-22 were plotted to determine the relative expression in normal and tumor tissue samples. 522 HNSCC patients were categorized into high and low miR-22 expression groups according to the median expression values of the miR-22 gene. The cut-off value of 50% was determined by the Maxstat method. GraphPad Prism software was used to generate the Kaplan–Meier curves and to calculate the *p*-value for overall survival by a two-tailed log-rank test.

### Cell culture and stable cell line generation

Normal human epidermal keratinocytes (NHEK), human immortalized keratinocytes (HaCaT), and human squamous cell carcinoma cell lines (Colo-16 and SCL-1) were obtained from the Cell Bank of the Chinese Academy of Sciences (Shanghai, China). Human squamous cell carcinoma cell line (A431) and HEK293T were purchased from the American Type Culture Collection (ATCC).These cells were cultured in Dulbecco’s Modified Eagle’s Medium (DMEM) (#C11995500CP, Gibco) containing 10% fetal bovine serum (#BISH0085, BI) and 1% penicillin/streptomycin. All cells were maintained in a humidified incubator equilibrated with 5% CO_2_ at 37 °C and were tested and confirmed to be free of mycoplasma infection. A detailed protocol for stable cell line generation is provided in the Supplementary Methods.

### Chemical skin carcinogenesis

For skin carcinogenesis, DMBA (#D3254, Sigma) and TPA (#P8139, Sigma) were dissolved in acetone and used as a carcinogen and a promoter, respectively. Mice were treated according to the two-stage carcinogenesis protocol [22]. The dorsal skin of female mice 7–9 weeks of age were shaved and after 48 h was initiated by topical application of 100 nmol of DMBA in 0.2 mL acetone. Two weeks after initiation, mice were treated topically twice weekly with 6.8 nmol TPA. Tumor multiplicity (average tumors per mouse), tumor incidence (percentage of mice with at least one tumor), and tumor size were recorded weekly for the remainder of the study.

### Real-time quantitative PCR

Total RNA was isolated using the mirVana^TM^ RNA Isolation kit following the manufacturer’s instructions (#AM1560, ThermoFisher Scientific). Each RNA sample was reverse-transcribed with the RevertAid First Strand cDNA Synthesis Kit (#K1621, ThermoFisher Scientific) using Oligo (dT) primers. However, *miR-22* and U6 were performed with designed reverse primer: *miR-22*-RT:GTCGTATCCAGTGCAGGGTCCGAGGTGCACTGGATACG.

ACACAGTTCT, U6-RT: AAAATATGGAACGCTTCACGAATTTGC. Relative quantitation was determined using the LightCycler 480 real-time PCR system (Roche) and then calculated by means of the comparative Ct method (2^−ΔΔCt^) with the expression of GAPDH as control. For microRNA expression, U6 snRNA was used as an internal control. The primers used were listed in Supplementary Table 1.

### RNAseq

Total RNA was isolated from A431 spheroid (2 Cas9-NC and 2 *miR-22* ko spheroids) using TRIzol Reagent (#15596026, Invitrogen) according to the manufacturer’s instructions. Then the RNA samples were submitted to the Berry Genomics Corporation and were sequenced on an Illumina NovaSeq 6000 platform. Genes that showed larger than 1.5-fold difference (FC > 1.5) in the relative mRNA abundance with *p* < 0.10 were considered differentially expressed.

### In vivo metastasis

Colo-16 cells that had been transfected to stably express firefly luciferase (pLV-luciferase) were infected with lentiviruses carrying empty vector with shctrl, *miR-22* KO, *miR-22* KO with shFOSB or shPAD2. These cells were injected into the lateral tail vein (5 × 10^5^ cells) of 5-week-old female BALB/C nude mice. For bioluminescence imaging, mice were injected with 200 mg/g of Beetle Luciferin, Potassium Salt (#E1603, Promega) in PBS. After injection for 5 min bioluminescence was imaged with a charge-coupled device camera (IVIS; Xenogen).

### Tumor treatment experiment

1.0 × 10^7^ A431 cells were subcutaneously implanted into the left and right flanks of 5-week-old female BALB/c nude mice. At 10 days after implantation, NC or *miR-22* antagomir (antagonist) (GenePharma, SUZHOU) were injected into the left or right tumor, respectively, and the mice have injected cisplatin intraperitoneally or not at the same time. The injection was repeated every other day. The experiments were performed “blind” with respect to the different treatments. The tumor diameters were measured and recorded every 3 days to generate a tumor growth curve. After 6 weeks of treatment, the tumors were excised and snap-frozen for RNA and protein extraction or paraffin-embedded for IHC staining.

### Statistical analysis

All data are expressed as the mean ± sd from three independent experiments, unless specified, each performed at least in triplicate. Statistical significance was evaluated with a two-tailed *t*-test by using SPSS 17.0 software. A *p*-value of <0.05 was considered significant.

### Supplementary information

Supplementary Methods

Supplementary Table 1

Supplementary Fig. 1

Supplementary Fig. 2

Supplementary Fig. 3

Supplementary Fig. 4

Supplementary Fig. 5

Supplementary Fig. 6

Supplementary Fig. 7

Supplementary Fig. 8

Supplementary Fig. 9

Supplementary Fig. 10

## Data Availability

RNAseq data that support the findings of this study have been deposited in the Gene Expression Omnibus under accession codes GSE156255.
