# Correction: *miR-22* promotes stem cell traits via activating Wnt/β-catenin signaling in cutaneous squamous cell carcinoma

**DOI:** 10.1038/s41388-023-02888-z

**Published:** 2023-11-17

**Authors:** Shukai Yuan, Peitao Zhang, Liqi Wen, Shikai Jia, Yufan Wu, Zhenlei Zhang, Lizhao Guan, Zhengquan Yu, Li Zhao

**Affiliations:** 1 Department of Biochemistry and Molecular Biology, School of Basic Medical Sciences, National Clinical Research Center for Cancer, Key Laboratory of Cancer Prevention and Therapy, Tianjin’s Clinical Research Center for Cancer, Tianjin Medical University Cancer Institute and Hospital, Tianjin Medical University, 22 Qixiangtai Road, Heping District, 300070 Tianjin, China; 2 Department of Nuclear Medicine, Tianjin Medical University General Hospital, 154 Anshan Road, Heping District, 300052 Tianjin, China; 3 State Key Laboratories for Agrobiotechnology, College of Biological Sciences, China Agricultural University, 2 Yuanmingyuan West Road, Haidian District, 100094 Beijing, China

**Keywords:** Cancer stem cells, Squamous cell carcinoma

Correction to: *Oncogene* 10.1038/s41388-021-01973-5, published online 3 August 2021

The authors found that there is a spelling mistake in the Abstract of this paper. The phrase “FosB-DDK1 transcriptional axis” should be corrected as “FosB-DKK1 transcriptional axis” in the sentence “Moreover, miR-22 also relieves DKK1-mediated repression of Wnt/β-catenin signaling by targeting a FosB-DDK1 transcriptional axis.”, which is in Line 5 to line 6 of the Abstract.

The original article has been corrected.”.

