# Supplementary material for: miR-22 promotes stem cell traits via activating Wnt/β-catenin signaling in cutaneous squamous cell carcinoma
Source: Oncogene. 2021 Aug 3;40(39):5799–813. doi: 10.1038/s41388-021-01973-5 (PMC8484012; doi:10.1038/s41388-021-01973-5)
Supplement: Supplementary file 1 — Supplementary Methods [file 41388_2021_1973_MOESM1_ESM.docx]

**Supplementary Methods**

**Stable Cell Line Generation**

The recombinant lentiviruses expressing *miR-22*, FOSB, PAD2, shFOSB and shPAD2 were constructed with expression vector PCDH-CMV-EF1α-puro and plko.1. For preparation of lentivirus, the core plasmids with lentivirus construction were co-transfected into HEK293T cells with the PAX8 and VSVG plasmids at a ratio of 10:3.5:6.5 by Lipofectamine 3000 (#L3000015, Invitrogen). The virus particles were harvested at 24 and 48 hours after transfection and filtered by 0.45μM filter unit (Millipore). A431 or Colo-16 cells were infected with lentivirus for 24 hours and stable clones were selected in 1μg/mL puromycin two days after infection. The shRNA targeted sequences were (sense sequences): FOSB #1: CTCTTTACACACAGTGAAGTT; FOSB #2: GCCGAGTCTCAATATCTGTCT; PAD2 #1: GCACCTTCATCGACGACATTT; PAD2 #2: AGGTCACCGTCAACTA-

CTATG. The scramble shRNA (CCTAAGGTTAAGTCGCCCTCG) was used as a negative control for FOSB or PAD2 knockdown cell lines. For miR-22, FOSB and PAD2 overexpression cell lines, the A431 or Colo-16 cells were infected with lentivirus constructed with PCDH-CMV-EF1α-puro empty vector as the negative control.

CRISPR/Cas9 gene targeting: Guide RNAs to target genes were designed using the Zhang laboratory web resource (http://crispr.mit.edu). The sgRNA encoding oligonucleotides were cloned into lentiCRISPR v2 vector (Plasmid #52961) using standard procedures and lentivirus particles were produced to knock out *miR-22*. The sgRNAs were designed as following: *miR-22* #1: GAGGGCAACAGTTCTTCAAC; *miR-22* #2: TTCAACTGGCAGCTTTAGCT. The scramble gRNA (CTTCCATGCGGCCCGTTCAA) was as a negative control. After screened by Puro for 14 days, the expression level of miR-22 in miR-22 knockout cell line and negative control cell line was checked by qPCR. The DNA fragment of targeting region was amplified and sequenced to find possible base deletions or mutations.

**Transwell and cell wound healing assay**

A431 and Colo-16 stable cell lines were suspended in serum-free DMEM media at a concentration of 1.0×10^6^/ml. Four hundred microliter cell suspension were placed to the upper chamber of transwell apparatus and six hundred microliter DMEM with 10% FBS were placed in culture well. After 24-48h, cells which pass through the membrane were then stained with crystal violet and counted.

A431 and Colo-16 cell lines were cultured in 3.5 cm well. When the cell density was ~90%, a single scratch was made with a pipette tip across the cell layer and after rinsing with PBS, the re-population of the denuded area monitored by capturing images every 12 hours at fixed positions.

**Spheroid formation assay**

A431 and Colo-16 cells were maintained in growth medium. Eighty percent confluent cultures were dissociated with trypsin followed by serum-dependent inactivation of the trypsin. The cells were collected by centrifugation and resuspended in spheroid medium consisting of DMEM/F12 (1:1) (#11330032, Gibco) containing 2% B27 serum-free supplement (#1750404, Gibco), 20ng/ml epidermal growth factor (#E4269, Sigma), 0.4% bovine serum albumin (#B4287, Sigma,) and 4 μg/ml insulin (#19278, Sigma) , and then were plated at 6-well ultra-low attachment culture dishes at 40 000 cells per well. Spheroids grew at different rates and increased in size with time. We defined spheroids as a collection of cells achieving a diameter ≥25 μm [1].

**Dual Luciferase Activity Assays**

To generate reporter constructs for luciferase assays, segments of about 650 bp in length containing predicted *miR-22* target sites in the 3’UTRs of *PAD2, FOSB* and *HOXA5* were cloned into the psiCHECK-2 vector (Promega) immediately downstream of the *Renilla* luciferase gene. To generate reporters with mutant 3’UTRs, six nucleotides (GCAGCT) in the target site complementary to the position 2–7 of the *miR-22* seed region were mutated to TAGATC by a QuikChange Site-Directed Mutagenesis kit according to the manufacturer’s protocol (Stratagene). 293T cells were seeded in 96-well plate one day before transfection. Ten nanograms of each reporter construct was co-transfected with *miR-22* mimic or a negative control at a final concentration of 50 nM into 293T cells using Lipofectamine 3000 according to the manufacturer’s protocol (Invitrogen). After 24 h, firefly and *Renilla* luciferase activities were measured with the Dual-Glo luciferase assay system according to the manufacturer’s instructions (Promega). All samples were analyzed in triplicates.

**TOP/FOPflash reporter assay**

TOP/FOPflash reporter assay was performed in a 24-well plate. For each well, A431 cells were co-transfected with 0.5ug PCDH or 0.5ug PCDH-*miR-22*, 0.5 μg TOPFlash or FOPflash and 0.05 μg PLRTK using Lipofectamine 3000. Both firefly and the renila luciferase activities were measured 24 h later by using Dual Luciferase Reporter Assay System Kit (#E2940; Promega) according to the recommended protocol. The TOPFlash or FOPFlash activity was normalized to Renilla luciferase signals.

**Histology, immunochemistry and immunofluorescence**

Immunochemistry and immunofluorescence were performed as described previously [2]. Tumor tissues were fixed in 4% PFA, paraffin-embedded and 5-μm sections were stained with hematoxylin and eosin (H&E). The sources of antibodies were K14 (#ab7800, Abcam), Cleaved Caspase-3 (#9661, CST), Ki67 (#ab16667, Abcam), GFP (#ab290, Abcam), Lgr5 (#ab75732, Abcam), PADI2 (#12110-1-AP, Proteintech), p63 (#ab172731, Abcam), FOSB (#2251, CST), VEGFR2(#9698), DKK1 (#21112-1-AP, Proteintech). Images were taken by Nikon ECLIPSE 90i Microscope.

**Isolation , culture and flow cytometric analysis of cSCC primary cells**

Tumor tissues were washed with PBS or DMEM and cut into small pieces about 1-2 mm in size. Minced tumor pieces were then transferred into a tube containing trypsin and incubated at 37°C for an hour. Add equal volume of DMEM/10% FBS and pass the suspension through a 70 μm cell strainer. Add more DMEM/10% FBS to the dish to recover all cells and tissue and pass through a 40-μm cell strainer. Centrifuge the cell suspension at 500×g for 5 min and plate out the recovered cells in keratinocyte medium and incubate at 37°C in a 5% CO_2_ incubator.

The isolated primary cells were stained with anti-Lgr5 antibody (#ab75732, Abcam) and secondary antibody, and then subjected to the BD FACSAria II flow cytometer.

**In situ hybridization**

MiRNA in situ hybridizations was performed by adopting the previously described protocol [3]. Double DIG-labeled *miR-22* and scrambled LNA probes (Exiqon) were hybridized at 55°C. In situ signals were detected by staining with Anti-Digoxigenin-AP antibody (Roche) and developing using BM purple substrate (Roche).

**Immunoprecipitation**

A431-PCDH (A431-NC) and A431-PCDH-*miR-22* (A431-*miR-22*) cells were treated with MG132(20uM) for 6 h and then were homogenized in ice-cold lysis buffer ( 50mM Tris-HCl pH 7.4, 150mM NaCl, 2mM EDTA, 5% NP-40) with 1% PMSF. Samples were centrifuged at 12,000g for 15 min at 4°C and the protein concentration was determined by the BCA (bicinchoninic acid) protein assay kit (#23225, Thermo Scientific). For single reaction, 30μl Protein A/G Agarose (#20421, Thermo Scientific) beads were incubated with 4μg β-catenin antibody for 6h at 4°C, followed by incubation with 1 mg protein lysis overnight. The beads were washed for 4 times with PBST, and then the samples were boiled with 2×loading buffer. Input samples and IP protein citrullination were determined by Immunoblotting using Anti-Citrulline (#17-347B, Millipore).

**GLUC reporter assay**

The GLUC reporter system was performed by adopting the previously described protocol [4]. HEK293T cells were co-transfected with different GLUC constructs of DKK1 promoter and FOSB overexpressed plasmid or control plasmid. After 48h, the luciferase activities were measured with the manufacturer’s instructions (Promega).

**Chromatin immunoprecipitation(ChIP)**

ChIPs were performed in cells as descried previously [4]. Briefly, A431 cells were cross-linked by 1% formaldehyde, sonicated, pre-cleared, and incubated with 4 μg FOSB antibody or normal IgG per reaction. Protein–DNA complexes were bound to Protein A/G Agrose beads, washed with different concentrations of salt buffers, then the pulled-down DNA was extracted, precipitated, and subjected to qPCR with specific primers. Exogenous ChIP was performed with Flag Agarose beads (#A2220, Sigma) in A431-pLV and A431-Flag-FOSB cell lines. The primers were used as follows: -1126-F: AGCCTTACTTTATTATATCCA, -1126-R: AATGCAGGGTTTGC

TCCCAAGTC; control-F: GACGCCTGTCATTTAAGAGCG, control-R: GGGGAA

AGCCATTTAGCCAAT.

**Xenograft mouse model**

Briefly, male NOD/SCID and nude mice (BALB/c nude, 4–5 weeks old) were used. Cas9-NC or *miR-22* KO spheroid A431 cells with number of 1.0×10^5^ were subcutaneously implanted into the left and right flanks of NOD/SCID mice. At 35 days after implantation, tumor pieces (~3 mm) from each group were inserted into the incision made in the dorsal skin of BALB/c nude mice. The tumor diameters were measured and recorded every three days to generate a tumor growth curve. After tumor growth assessment, the tumors were excised and snap frozen for RNA and protein extraction or paraffin-embedded for IHC staining.

Supplementary References

1. Adhikary G, Grun D, Balasubramanian S, Kerr C, Huang JM, Eckert RL. Survival of skin cancer stem cells requires the Ezh2 polycomb group protein. Carcinogenesis. 2015;36:800-810.

2. Yuan S, Li F, Meng Q, Zhao Y, Chen L, Zhang H, et al. Post-transcriptional Regulation of Keratinocyte Progenitor Cell Expansion, Differentiation and Hair Follicle Regression by miR-22. PLoS Genet. 2015;11:e1005253.

3. Jørgensen S, Baker A, Møller S, Nielsen BS. Robust one-day in situ hybridization protocol for detection of microRNAs in paraffin samples using LNA probes. Methods. 2010;52:375-81.

4. Li X, Ruan X, Zhang P, Yu Y, Gao M, Yuan S, et al. TBX3 promotes proliferation of papillary thyroid carcinoma cells through facilitating PRC2-mediated p57 KIP2 repression. Oncogene. 2018;37:2773-2792.
