# Supplementary figures and images for: miR-22 promotes stem cell traits via activating Wnt/β-catenin signaling in cutaneous squamous cell carcinoma
Source: Oncogene. 2021 Aug 3;40(39):5799–813. doi: 10.1038/s41388-021-01973-5 (PMC8484012; doi:10.1038/s41388-021-01973-5)

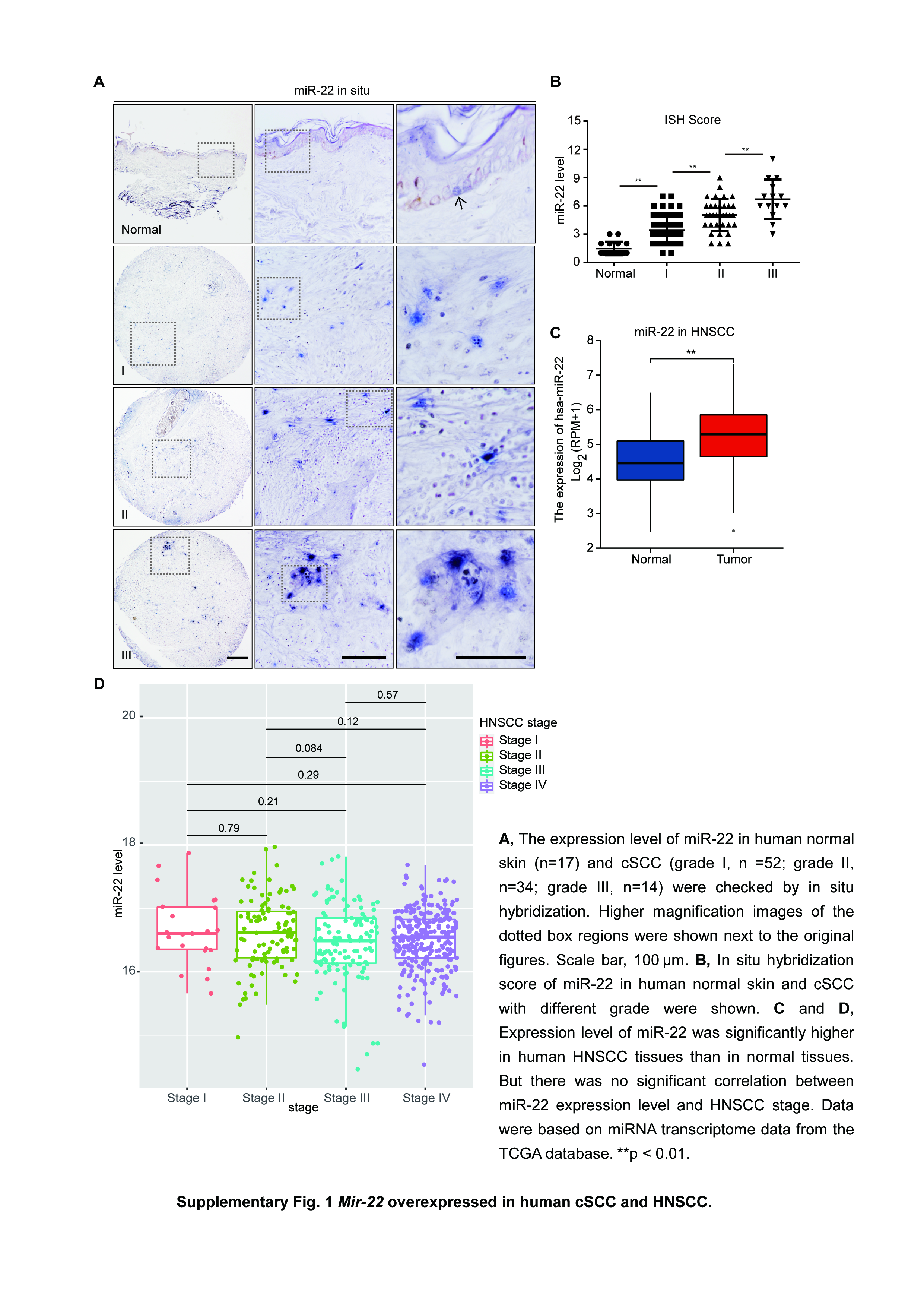

Supplement: Supplementary file 3 — Supplementary Fig. 1 [file 41388_2021_1973_MOESM3_ESM.tif]

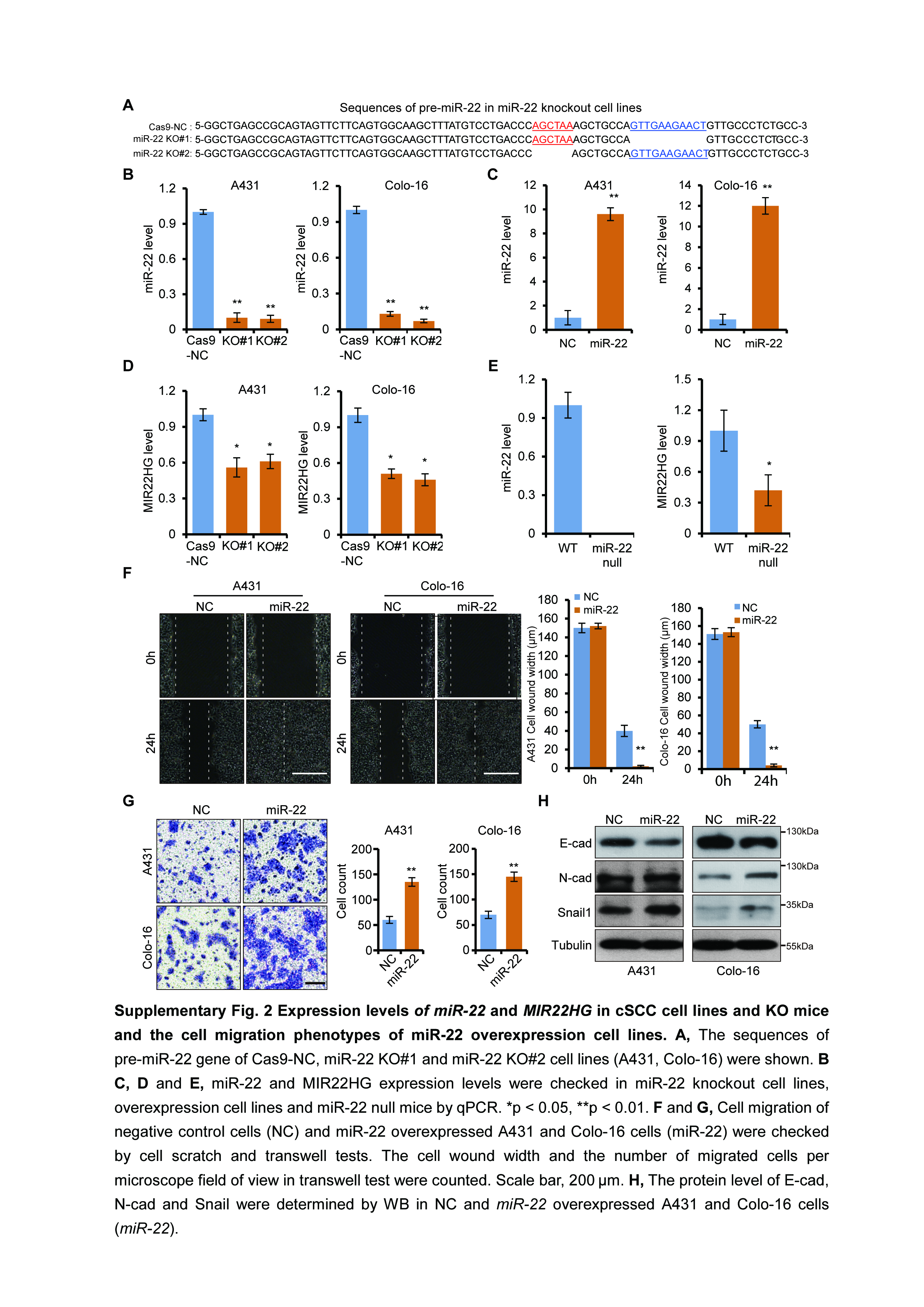

Supplement: Supplementary file 4 — Supplementary Fig. 2 [file 41388_2021_1973_MOESM4_ESM.tif]

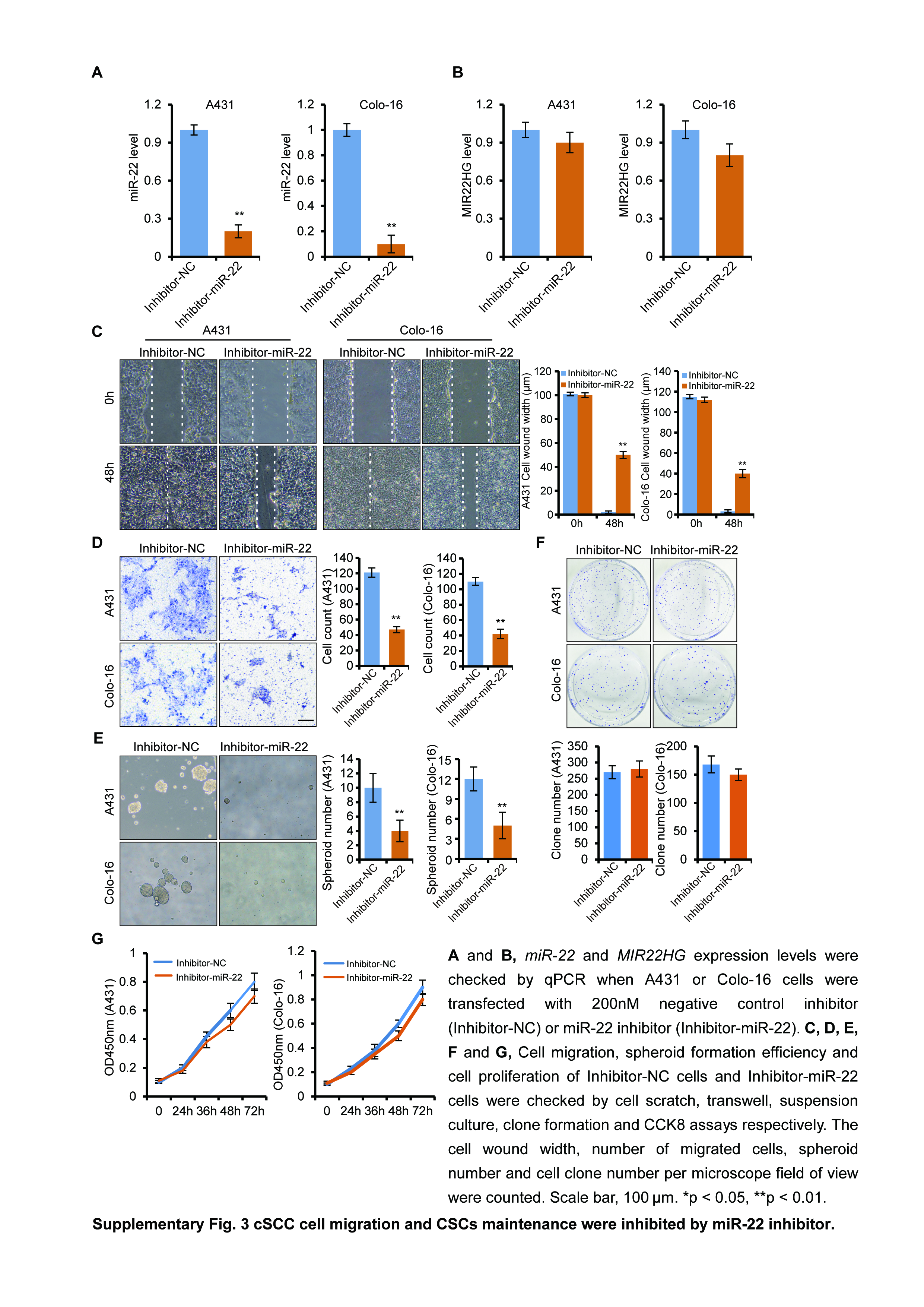

Supplement: Supplementary file 5 — Supplementary Fig. 3 [file 41388_2021_1973_MOESM5_ESM.tif]

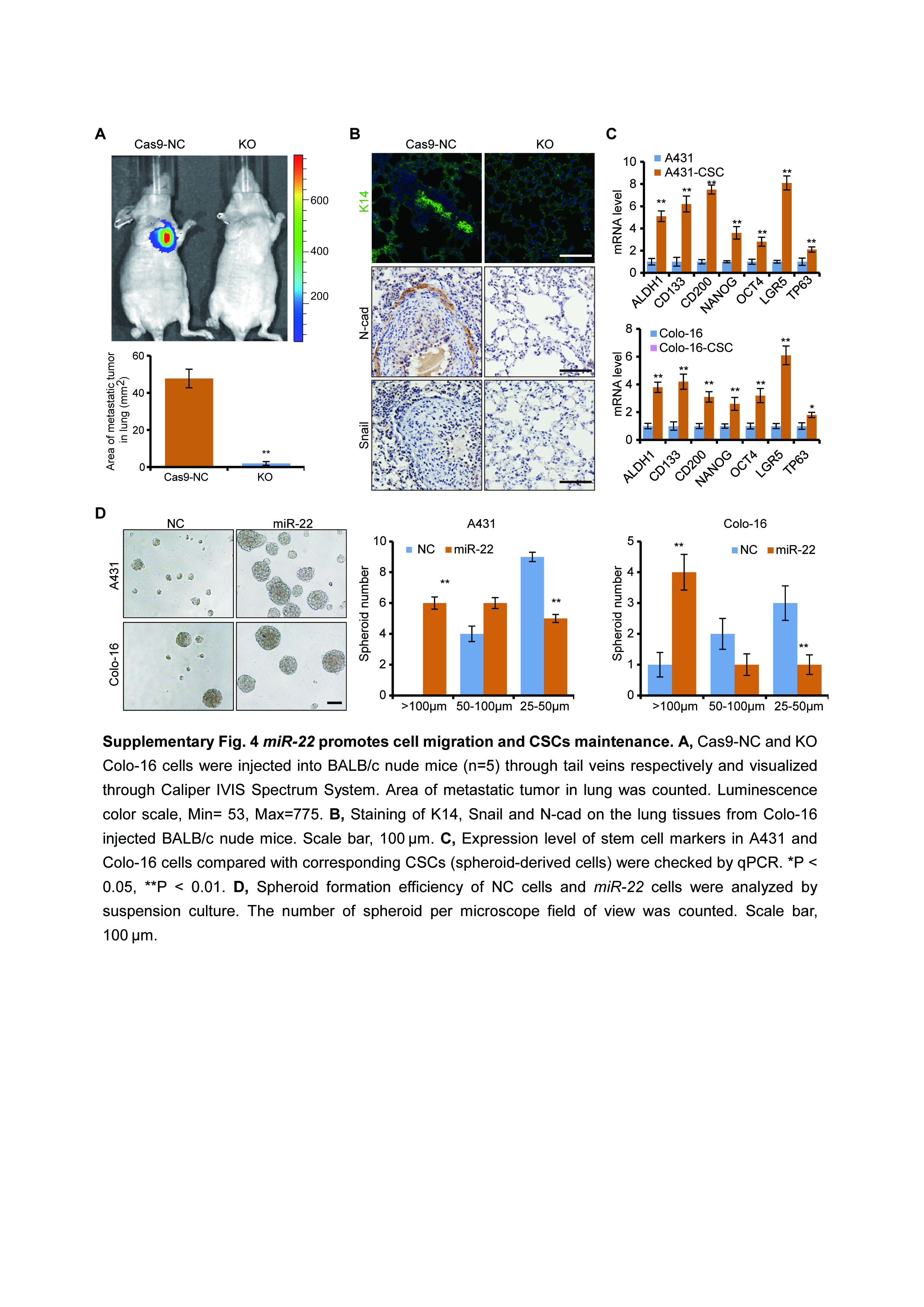

Supplement: Supplementary file 6 — Supplementary Fig. 4 [file 41388_2021_1973_MOESM6_ESM.tif]

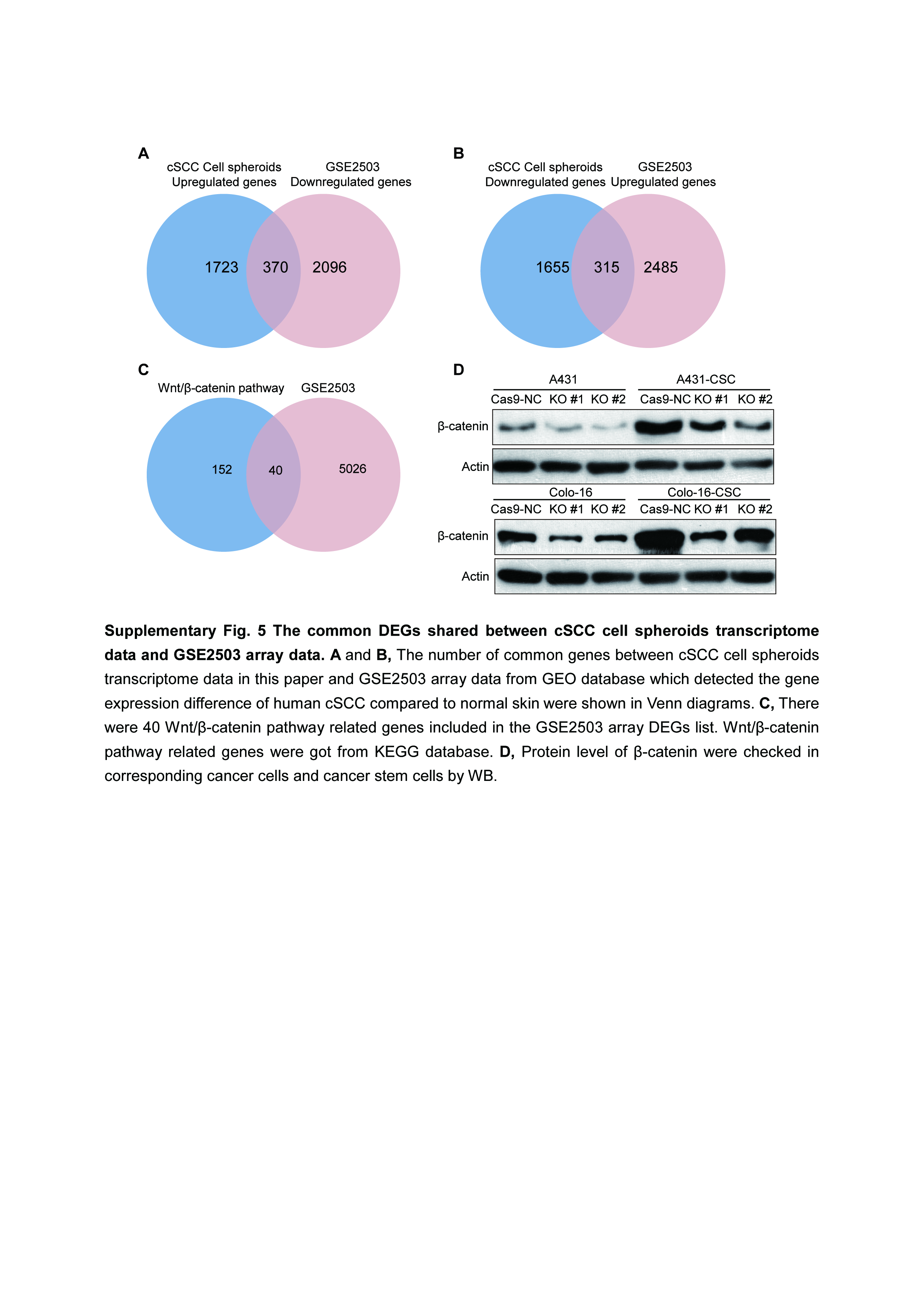

Supplement: Supplementary file 7 — Supplementary Fig. 5 [file 41388_2021_1973_MOESM7_ESM.tif]

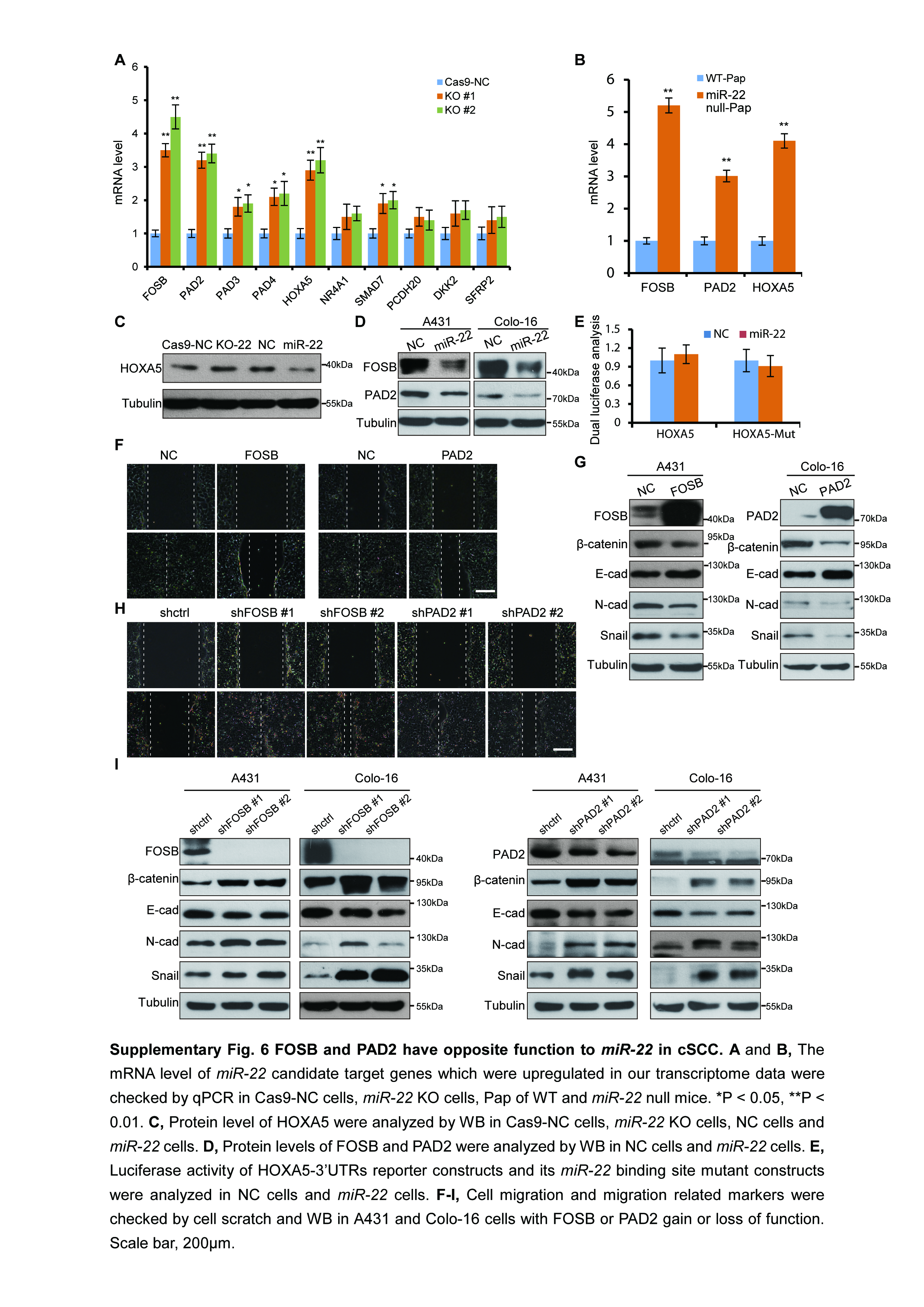

Supplement: Supplementary file 8 — Supplementary Fig. 6 [file 41388_2021_1973_MOESM8_ESM.tif]

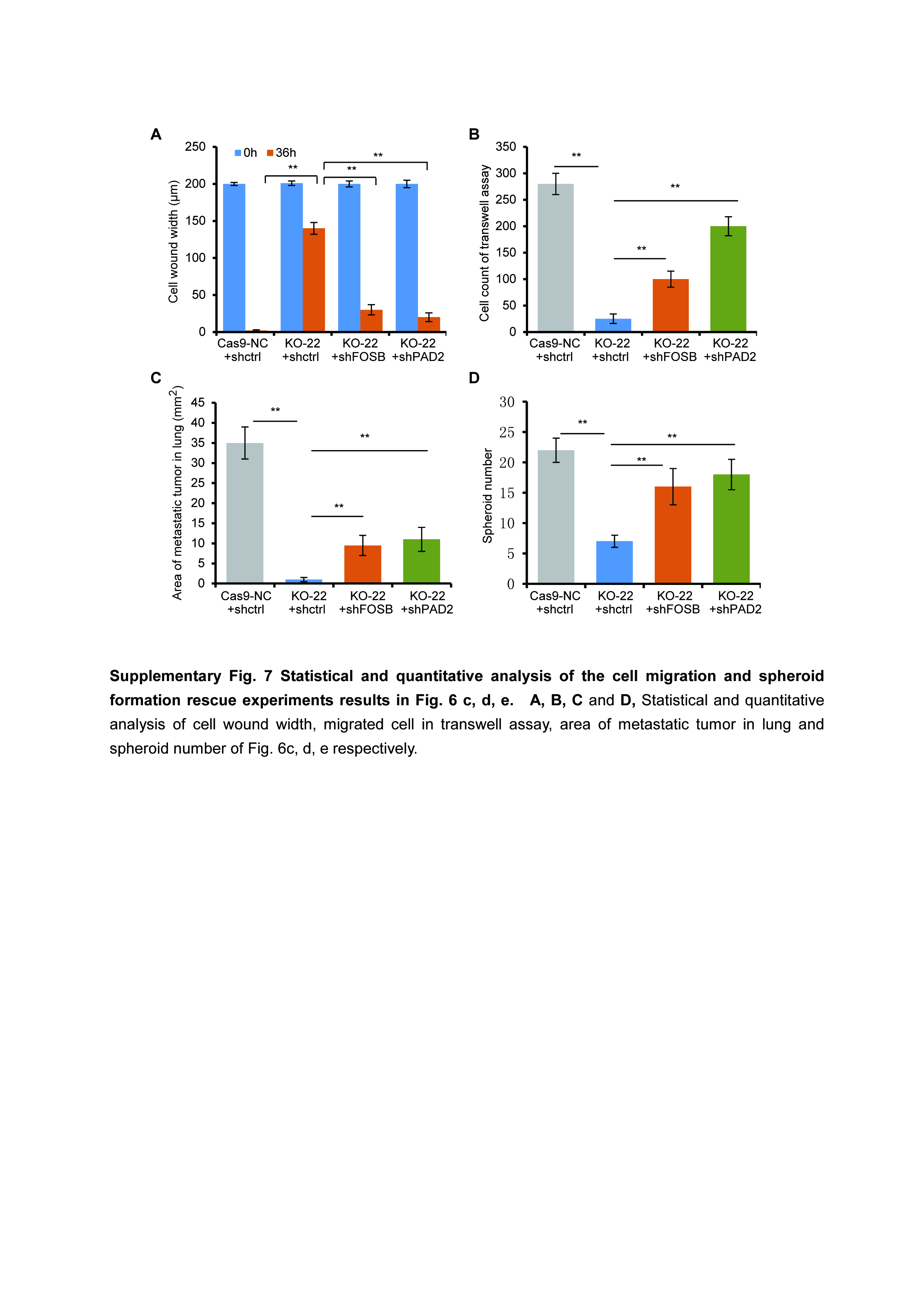

Supplement: Supplementary file 9 — Supplementary Fig. 7 [file 41388_2021_1973_MOESM9_ESM.tif]

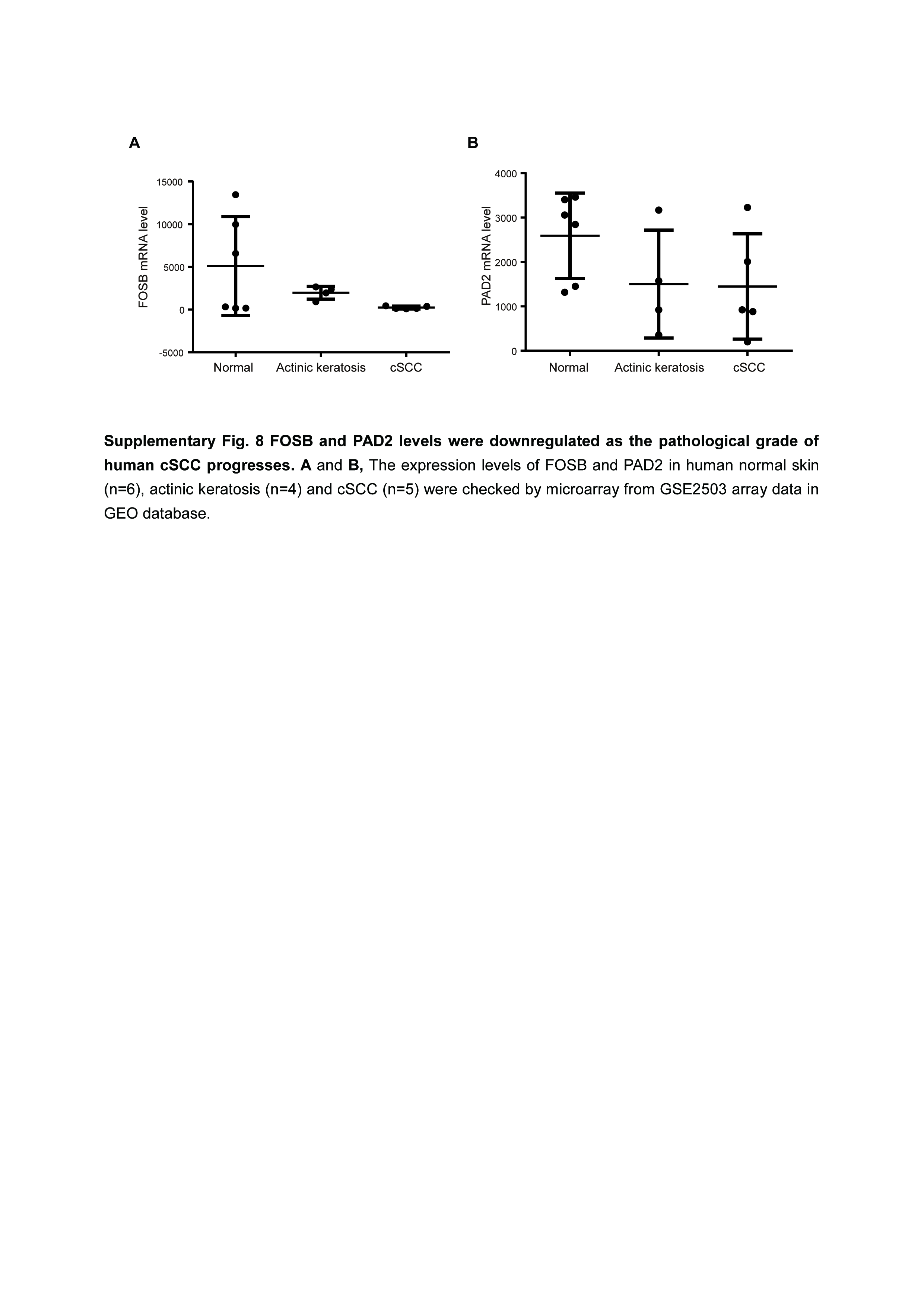

Supplement: Supplementary file 10 — Supplementary Fig. 8 [file 41388_2021_1973_MOESM10_ESM.tif]

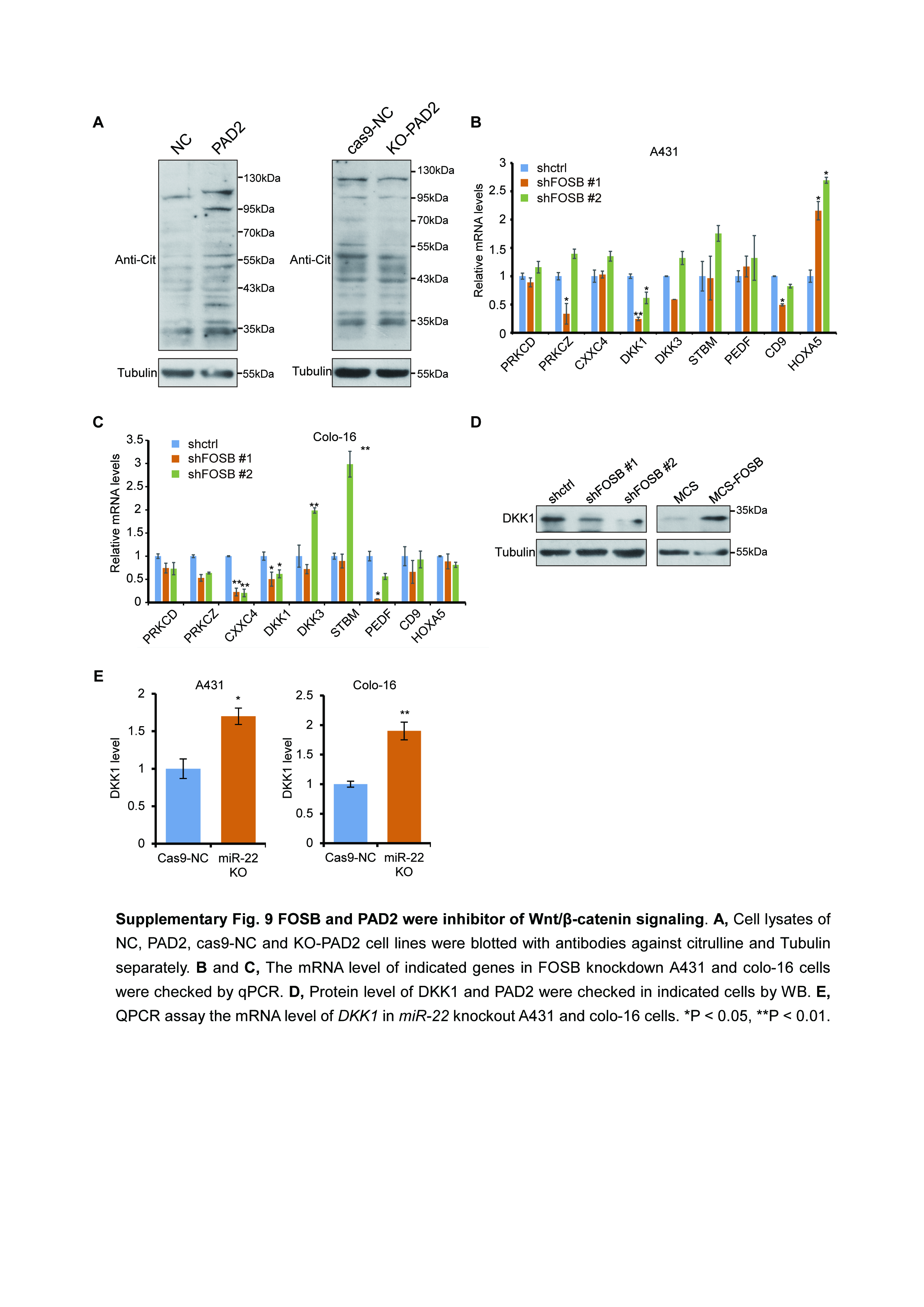

Supplement: Supplementary file 11 — Supplementary Fig. 9 [file 41388_2021_1973_MOESM11_ESM.tif]

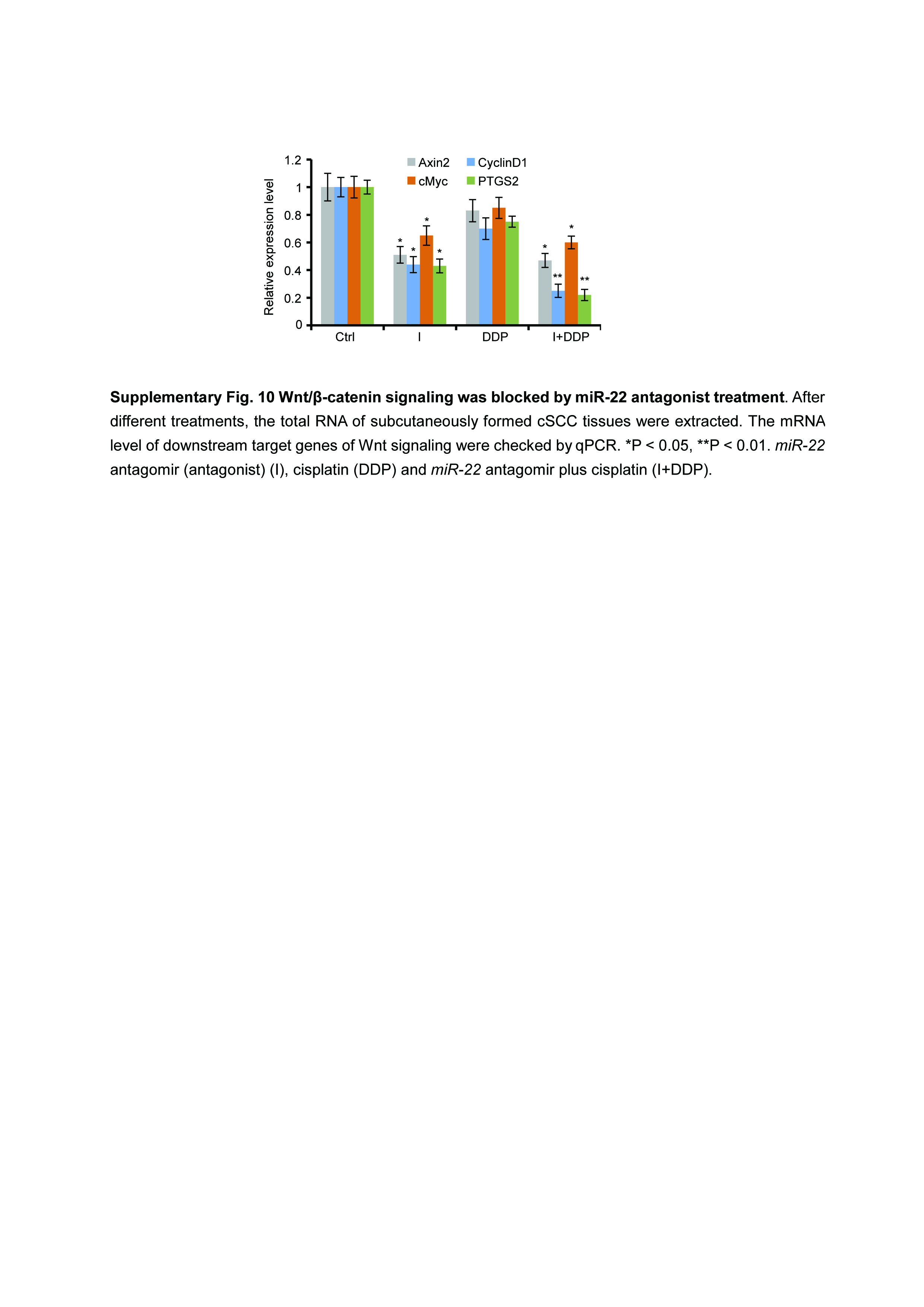

Supplement: Supplementary file 12 — Supplementary Fig. 10 [file 41388_2021_1973_MOESM12_ESM.tif]
