# Supplementary figures and images for: Correction: miR-22 promotes stem cell traits via activating Wnt/β-catenin signaling in cutaneous squamous cell carcinoma
Source: Oncogene. 2022 Feb 4;41(11):1674–6. doi: 10.1038/s41388-022-02188-y (PMC8913364; doi:10.1038/s41388-022-02188-y)

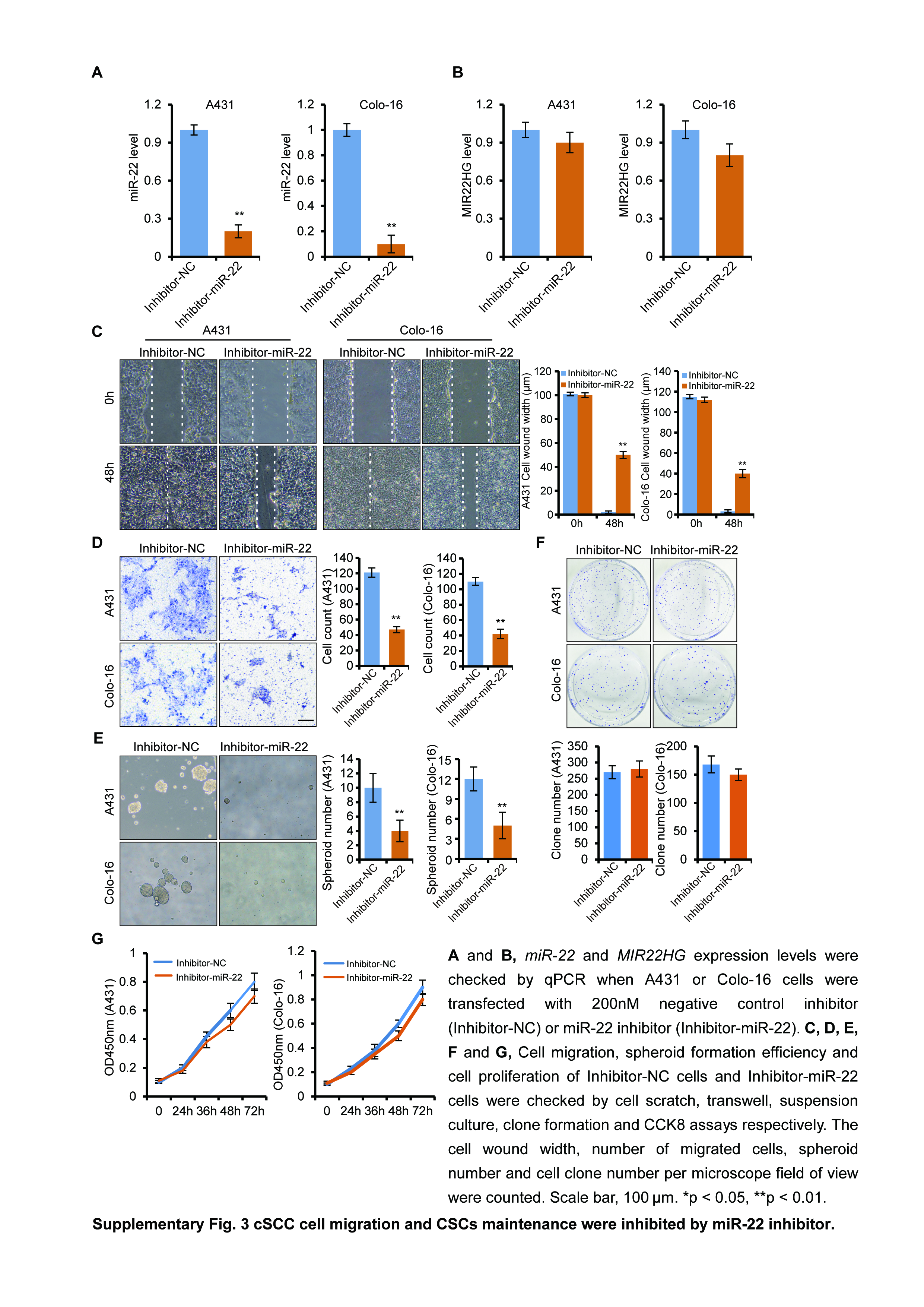

Supplement: Supplementary file 1 — Supplementary Fig. 3 [file 41388_2022_2188_MOESM1_ESM.tif]
